# Cloning, expression, and *in silico* structural modeling of cholesterol oxidase of *Acinetobacter* sp. strain RAMD in *E. coli*

**DOI:** 10.1002/2211-5463.13254

**Published:** 2021-07-31

**Authors:** Hoda E. Mahmoud, Shaymaa W. El‐Far, Amira M. Embaby

**Affiliations:** ^1^ Department of Biotechnology Institute of Graduate Studies and Research Alexandria University Egypt; ^2^ Division of Pharmaceutical Microbiology Department of Pharmaceutics and Industrial Pharmacy College of Pharmacy Taif University Saudi Arabia

**Keywords:** *Acinetobacter* sp., choxAB, class I cholesterol oxidase, cloning, heterologous expression, structural modeling

## Abstract

Cholesterol oxidases (CHOXs) are flavin‐adenine dinucleotide‐dependent oxidoreductases with a range of biotechnological applications. There remains an urgent need to identify novel CHOX family members to meet the demands of enzyme markets worldwide. Here, we report the cloning, heterologous expression, and structural modeling of the cholesterol oxidase of *Acinetobacter* sp. strain RAMD. The cholesterol oxidase gene was cloned and expressed in pGEM®‐T and pET‐28a(+) vectors, respectively, using a gene‐specific primer based on the putative cholesterol oxidase ORF of *Acinetobacter baumannii* strain AB030 (GenBank [gb] locus tag: IX87_05230). The obtained nucleotide sequence (1671 bp, gb: MK575469.2), translated to a protein designated choxAB (556 amino acids), was overexpressed as inclusion bodies (IBs) (MW ˜ 62 kDa) in 1 mm IPTG‐induced *Escherichia coli* BL21 (DE3) Rosetta cells. The optimized expression conditions (1 mm IPTG with 2% [v/v] glycerol and at room temperature) yielded soluble active choxAB of 0.45 U·mL^−1^, with 56.25‐fold enhancement. The recombinant choxAB was purified to homogeneity using Ni^2+^‐affinity agarose column with specific activity (0.054 U·mg^−1^), yield (8.1%), and fold purification (11.69). Capillary isoelectric‐focusing indicated pI of 8.77 for choxAB. LC‐MS/MS confirmed the IBs (62 kDa), with 82.6% of the covered sequence being exclusive to *A. baumannii* cholesterol oxidase (UniProtKB: A0A0E1FG24). The 3D structure of choxAB was predicted using the LOMETS webtool with the cholesterol oxidase template of *Streptomyces* sp. SA‐COO (PDB: 2GEW). The predicted secondary structure included 18 α‐helices and 12 β‐strands, a predicted catalytic triad (E^220^, H^380^, and N^514^), and a conserved FAD‐binding sequence (GSGFGGSVSACRLTEKG). Future studies should consider fusion to solubilization tags and switching to the expression host *Pichia pastoris* to reduce IB formation.

## Graphical abstract

The cholesterol oxidase (choxAB) encoding ORF (1671 bp & 556 a.a) of *Acinetobacter* sp. strain RAMD was cloned (pGEM®‐T vector) and overexpressed (pET‐28a(+) vector) in *E. coli* BL21(DE3) Rosetta cells as inclusion bodies for the first time. Optimized production for recombinant soluble choxAB yields active choxAB (0.45 U·mL^−1^). The purified recombinant choxAB (62 kDa) showed 0.054 U·mg^−1^ and 11.69‐fold purification.

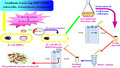

**Abbreviations**
CHOXs: cholesterol oxidases
cIEF: capillary‐imaged capillary isoelectric focusing
DTT: dithiothreitol
EDTA: ethylene‐diamine tetra‐acetic acid
FAD: flavin‐adenine dinucleotide
IAA: iodoacetamide
IBs: inclusion bodies
IPTG: isopropyl β‐d‐1‐thiogalactopyranoside
LC‐MS‐MS: liquid chromatography with tandem mass spectrometry
LOMETS: Local Meta‐Threading Server
MALDI-TOF-MS: matrix‐assisted laser desorption ionization time‐of‐flight mass spectrometry
ORF: open reading frame
PDB: Protein Data Bank
pI: isoelectric point
PMSF: phenylmethylsulfonyl fluoride
RMSD: root mean square deviation
X‐gal: 5‐bromo‐4‐chloro‐3‐indolyl‐β‐d‐galactopyranoside

Cholesterol oxidases (CHOXs), namely, 3β‐hydroxysteroid oxidases (EC1.1.3.6), are flavin‐adenine dinucleotide (FAD)‐dependent oxidoreductases that have received much attention in recent years. They display a wide range of biotechnological applications [1, 2, 3, 4] in numerous fields, including diagnostics (e.g., determination of cholesterol levels in patient serum and food samples), pharmaceuticals (bioconversion of several sterols and nonsteroidal alcohols), and agriculture (biological insecticides). CHOXs display dual oxidase and isomerase activities, first catalyzing cholesterol oxidation to its derivative cholest‐5‐en‐3‐one and then isomerizing that to cholest‐4‐en‐3‐one with the liberation of H_2_O_2_ as a by‐product [5, 6]. Typically, CHOXs have two main domains: the FAD‐binding and substrate‐binding domains. The FAD cofactor can bind to the enzyme either covalently or noncovalently; hence, CHOXs comprise two classes: class I with noncovalent binding and class II with covalent binding [7].

CHOX enzymes are exclusively sourced from bacteria, both pathogenic and nonpathogenic; no homologs have been discovered in eukaryotes to date. Nonpathogenic bacteria employ CHOXs mainly to obtain carbon and energy sources from the degradation of cholesterol, while pathogenic bacteria utilize CHOXs to invade host macrophages by oxidizing membrane cholesterol [8, 9]. Both native and recombinant CHOXs have been produced, purified, and characterized from a panel of bacterial species, including *Bacillus* spp., *Streptomyces* spp., *Serratia marcescens* W8, *Brevibacterium* spp., *Arthrobacter simplex*, *Chromobacterium* sp. DS‐1, *Castellaniella* sp., *Chryseobacterium gleum*, *Corynebacterium cholesterolicum*, *Mycobacterium tuberculosis*, *Rhodococcus* spp., *Burkholderia* spp., and *Bordetella* sp. [10]. However, there remains an urgent need to search for novel CHOX family members to meet the demands of enzyme markets worldwide.

*Acinetobacter baumannii* is a Gram‐negative bacterial pathogen primarily associated with health implications, particularly among immunocompromised hospital patients [11]. Numerous full genome sequences of several *A. baumannii* strains have been published in GenBank (gb), and their study has yielded putative open reading frames (ORFs) of *chox* genes. However, no reports have highlighted the production of CHOX from *A. baumannii*; studying endogenous CHOX in *A. baumannii* requires strict laboratory safety precautions to avoid the unwanted health consequences of handling a biosafety level 2 pathogen. The typical alternative and eco‐friendly approach would be to clone and express *A. baumannii*’s *chox* gene in the heterologous expression host *Escherichia coli*. This approach is commonly used in the large‐scale manufacturing of recombinant proteins.

Accordingly, the present study aimed to unravel the nature of the putative *chox*‐encoding gene in the locally isolated clinical *Acinetobacter* sp. strain RAMD through its cloning, *in silico* analysis, and expression in *E. coli* for the first time ever.

## Methods

### Bacterial strains and vectors

A local clinical isolate of *Acinetobacter* sp. was obtained from the Faculty of Medicine’s Microbiology Department in Alexandria University, Egypt. It was used as the source of the CHOX open reading frame (ORF). *E. coli* strains DH5α (Promega Co., Madison City, WI, USA) and BL21 (DE3) Rosetta (Novagen Co., Madison City, WI, USA) were used as propagation and expression hosts, respectively. pGEM®‐T‐Easy (Promega Co., Madison City, WI, USA) and pET‐28a(+) (GenScript Co., Piscataway, NJ, USA) were used as cloning and expression vectors, respectively.

### Genomic DNA isolation

The genomic DNA of *Acinetobacter* sp. was isolated using the ZR Fungal/Bacterial DNA Miniprep^TM^ Kit (Zymo Co., Irvine, CA, USA) according to the manufacturer’s instructions. The concentration of the isolated DNA was estimated using a NanoDrop™ 2000/2000c spectrophotometer.

### Validation of the *Acinetobacter* sp. identity

#### 16S rDNA sequence analysis

The molecular identification of the clinical isolate was carried out using a 16S ribosomal DNA (rDNA) sequence analysis. The full length of the *16S rDNA* gene was amplified using the universal 16S rDNA primer set of 27 F (5′‐AGAGTTTGATCCTGGCTCAG‐3′) and 1492 R (5′‐GGTTACCTTGTTACGACTT‐3′) [12], and the polymerase chain reaction (PCR) was performed as described previously [13]. PCR products were purified using the GeneJET PCR Purification Kit (Thermo Fisher Scientific Co., Waltham, MA, USA) according to the manufacturer’s instructions, and the purified PCR products were directly sequenced using ABI PRISM BigDye™ Terminator Cycle kits with the 27F and 1492R primer set. The obtained 16S rDNA nucleotide sequence was then matched against international nucleotide databases (e.g., GenBank, the European Molecular Biology Laboratory [EMBL], and the DNA Data Bank of Japan [DDBJ]) through the Basic Local Alignment Search Tool (BLASTN) of the National Center for Biotechnology Information (NCBI). A phylogenetic tree was constructed using clc sequence viewer 8.0 (Aarhus City, Denmark) to determine the evolutionary relationship of the obtained isolate sequence with sequences from other related bacteria. The obtained 16S rDNA nucleotide sequence was additionally deposited in the GenBank database (accession MK879410.1).

#### MALDI‐TOF MS identification

The species identity of the clinical isolate was additionally evaluated through matrix‐assisted laser desorption ionization time‐of‐flight mass spectrometry (MALDI‐TOF MS) (Bruker Daltonics, Leipzig, Germany). This analysis was conducted as described previously [14] and according to the instructions given in the mass spectrometer user manual. Raw spectra were processed using the MALDI Biotyper 2.0 software (Bruker Daltonics).

### Isolation of the cholesterol oxidase gene

The putative ORF sequence encoding the cholesterol oxidase gene of the *A. baumannii* strain AB030 was retrieved from GenBank (locus tag: IX87_05230) and used to design a gene‐specific primer set:

F‐*choxAB‐full length* (5’‐GAATTCATGACAATAAACAATTATGACTATG‐3’) and
R‐*choxAB‐full length* (5’‐CTCGAGAGCAGATACTTTTGCTGCTTCTA‐3’).

The underlined sequences in each primer indicate recognition sites for the restriction enzymes *Eco*RI and *Xoh1*, respectively. PCR was performed in a 50 μL total volume containing genomic DNA (50 ng), a 25 μL 2X PCR Master Mix solution (MyTaq™ Mix, Bioline, London, UK), forward and reverse primers (0.5 μm each), and nuclease‐free water. The PCR conditions were as follows: 95 °C for 5 min, 30 cycles each with 94 °C for 1 min, 55 °C for 1 min, and 72 °C for 100 s, and then a final extension at 72 °C for 10 min.

### Cloning and sequencing of the cholesterol oxidase gene

The amplified PCR product was designated as *choxAB* and purified using the GeneJET PCR Purification Kit (Thermo Fisher Scientific Co.) according to the manufacturer’s instructions. The *choxAB* fragment was cloned into the pGEM®–T‐Easy vector, with the ligation reaction carried out at 4 °C for 16 h in a total volume of 10 μL: 1 µL (50 ng) pGEM®–T‐Easy vector, 3 µL (150 ng) purified PCR product, 5 µL 2x ligation buffer, and 1 µL (2 Units) T4‐DNA ligase (Promega Co.). The pGEM®‐T/*choxAB* construct was then transformed into chemically competent *E. coli* DH5α cells [15]. The transformants were blue‐white screened on LB/ampicillin/IPTG (isopropyl β‐d‐1‐thiogalactopyranoside)/X‐gal (5‐bromo‐4‐chloro‐3‐indolyl‐β‐d‐galactopyranoside) plates [15], and the recombinant plasmids were isolated using the GeneJET Plasmid Miniprep Kit (Thermo Fisher Scientific Co.). The presence of the *choxAB* fragment in the recombinant plasmids was verified by PCR using the gene‐specific primer set (F‐*choxAB* and R‐*choxAB*) and by plasmid sequencing using ABI PRISM BigDye™ Terminator Cycle kits with the universal primer set (Sp6 and T7).

### Subcloning of *choxAB* into the pET‐28a(+) vector

The pGEM®‐T/*choxAB* construct was subcloned into the pET‐28a(+) expression vector by GenScript Co., using *EcoR1* and *Xho1* restriction enzymes. The GenScript construct #U3326EL100_1 is henceforth referred to as pET‐28a(+)/*choxAB*.

### Transformation of pET‐28a(+)/*choxAB* into *E. coli* strain BL21 (DE3) Rosetta

The pET‐28a (+)/*choxAB* expression construct was transformed into chemically competent *E. coli* BL21 (DE3) Rosetta cells [15] and the transformants grown on Luria–Bertani (LB)/kanamycin (34 μg·mL^−1^) plates.

### Production of recombinant *choxAB* in *E. coli* strain BL21 (DE3) Rosetta

For the *E. coli* culture, 200 mL of LB, supplemented with kanamycin at a final concentration of 34 µg·mL^−1^, was dispensed in each of the three 1‐L Erlenmeyer flasks, each of which was inoculated with a 2 mL overnight culture of recombinant *E. coli* BL21 (DE3) Rosetta cells harboring the pET‐28(a)+/*choxAB* construct. The cultures were allowed to grow at 37 °C for 3 h with an agitation speed of 180 rpm, achieving an optical density of 0.6–0.8 at 600 nm. Then, IPTG at a final concentration of 1 mm was added to each culture. The induced cultures were incubated at 37 °C, 30 °C, and room temperature as appropriate, with agitation at 180 rpm for 18 h more. After the incubation, the cells were pelleted from each induced culture separately by centrifugation at 5376 ***g*** at 4 °C for 20 min. The cell pellets were then suspended in 5 mL of a disruption buffer (50 mm Tris/HCl, pH 7.6; 50 mg·mL^−1^ lysozyme; and 300 mm NaCl) and incubated at 37 °C in a water bath for 30 min. Cell breakage was achieved by sonication at 14 000 Hz (Fisher Brand™ Sound Enclosure, Thermo Fisher Scientific Co.) for five cycles of 25 s each, with an interval of 20 min on ice between consecutive cycles. The cell debris was removed by centrifugation at 8400 ***g*** for 15 min at 4 °C, and the soluble supernatants were moved to new Eppendorf tubes. Both clear supernatants and insoluble precipitates (inclusion bodies) were preserved at −20 °C until further processing.

### Optimized production of recombinant active choxAB in *E. coli* strain BL21 (DE3) Rosetta

In order to obtain recombinant, soluble, active choxAB, the next procedures were followed: (a) expression and induction with five IPTG concentrations (0.2, 0.4, 0.6, 0.8, and 1 mm) from recombinant *E. coli* (BL21) DE3 Rosetta harboring the pET‐28(a)+/choxAB construct; (b) expression and induction with 1 mm IPTG under three different temperatures (room temperature, 30 °C, and 37 °C) from recombinant *E. coli* (BL21) DE3 Rosetta harboring the pET‐28(a)+/choxAB construct; (c) expression and induction with 1 mm IPTG under room temperature using four different growth media (2xTY, LB, M9 [minimal salts], and 5xLB); (d) expression and induction with 1 mm IPTG under room temperature by co‐adding the following additives together with 1 mm IPTG at the time of induction (cultures with optical density of 0.6–0.8 at 600 nm) [16]: (a) glycerol (1%, 2%, 3%, 4%, and 5% [v/v]), (b) sorbitol (0.1, 0.2, 0.3, and 0.4 m), (c) ethanol (1%, 2%, and 3% [v/v]), (d) glucose (5, 10, and 15 mm); (e) performing the induction at a low agitation speed of 100 rpm; (f) treating the insoluble recombinant expressed choxAB with four solubilization buffers (B, C, E, and F) according to a procedure previously mentioned [17]: (B) 50 mm Tris/HCl, 5 mm ethylene‐diamine tetra‐acetic acid (EDTA), 1 mm phenylmethylsulfonyl fluoride (PMSF), 6 m guanidine‐HCl, pH 8.5, (C) 50 mm Tris/HCl, 5 mm EDTA, 1 mm PMSF, 2 m urea, pH 12, (E) 50 mm Tris/HCl, 5 mm EDTA, 1 mm PMSF, 6 m *n*‐propanol, 2 m urea, pH 8.5, and (F) 50 mm Tris/HCl, 5 mm EDTA, 1 mm PMSF, 6 m *B*‐mercaptoethanol, 2 m urea, pH 8.5; and (g) treating the insoluble recombinant expressed choxAB with SDS‐KCl according to a previously reported procedure [18]. The resulting solubilized choxAB protein fractions from the treatment with the above‐mentioned solubilization buffers and SDS were dialyzed separately using a dialysis membrane of 14 000 Da against 50 mm Tris/HCl, pH 8.5, with a buffer change four times in 48 h.

### Purification of recombinant choxAB: a preliminary experiment

A culture of recombinant *E. coli* cells (500 mL), induced with 1 mm IPTG with the addition of 2% (v/v) glycerol at the time of induction, was prepared. After sonication using the above‐mentioned conditions, the soluble fraction of cell lysate containing 80 mg crude protein was subjected to partial purification on a small scale using a 1 mL Ni^2+^‐NTA affinity matrix in a batch mode. Unbound proteins were eliminated by washing the column with an equilibration buffer (50 mm phosphate buffer, pH 7.5, 10 mm imidazole, 300 mm NaCl). The elution of bound His‐tagged recombinant choxAB was performed by washing the column with an elution buffer (50 mm phosphate buffer, pH 7.5, 500 mm imidazole, 300 mm NaCl). The eluted fractions showing protein content (evidenced by absorbance at 280 nm) and a cholesterol oxidase activity were pooled and kept at 4 °C until they were processed further.

### Protein determination

The protein content in the crude sonicated cell extracts was estimated via the Bradford procedure using Coomassie Brilliant Blue G250 [19]. A standard curve was established using bovine serum albumin.

### Sodium dodecyl sulfate/polyacrylamide gel electrophoresis

The recombinant choxAB enzyme was subjected to 10% sodium dodecyl sulfate/polyacrylamide gel electrophoresis (SDS/PAGE) according to the Laemmli method [20]. The gel was stained with silver stain. The molecular mass of choxAB was determined using the GangNam‐STAIN™ Prestained Protein Ladder (iNtRON CO., Seongnam, Korea).

### LC‐MS‐MS

Liquid chromatography with tandem mass spectrometry (LC‐MS‐MS) was performed on the choxAB enzyme inclusion bodies at PhenoSwitch Bioscience, Canada.

#### Sample preparation

The crude inclusion bodies were re‐suspended in 2% SDS and loaded on a 4%–12% gradient gel for SDS/PAGE. The major visible band, at approximately 62 kDa, was cut out, dehydrated in 50% acetonitrile, and rehydrated in 50 mm Tris (pH 8.0) + 10 mm dithiothreitol (DTT). The sample was reduced for 15 min at 65 °C, after which cysteines were alkylated through the addition of 15 mm iodoacetamide (IAA) and incubated for 30 min in the dark at room temperature. The remaining IAA was then quenched by the addition of 10 mm DTT. The gel piece was dehydrated once more with 50% acetonitrile and rehydrated in a trypsin/LysC solution, with digestion overnight at 37 °C. The peptides were finally purified through reversed‐phase extraction and analyzed by liquid chromatography–mass spectrometry (LC‐MS).

#### Mass spectrometry

Data acquisition was performed with an ABSciex TripleTOF 6600 (ABSciex, Foster City, CA, USA) equipped with an electrospray interface with a 25 μm iD capillary and coupled with an Eksigent μUHPLC (Eksigent, Redwood City, CA, USA). The analyst tf 1.8 software (SCIEX, Framingham City, MA, USA) was used for instrument control, data acquisition, and data processing. Acquisition was performed in the information‐dependent acquisition (IDA) mode. The source voltage was set to 5.5 kV, the temperature maintained at 325 °C, the curtain gas set at 27 psi, and gas one and two set at 27 and 10 psi, respectively. Separation was performed on a reversed‐phase HALO C18‐ES column (0.3 μm i.d., 2.7 μm particles, 150 mm long) (Advance Materials Technology, Wilmington, DE), which was maintained at 60 °C. Samples were injected by loop overfilling into a 5 μL loop. For the 15‐min LC gradient, the mobile phase consisted of solvent A (0.2% v/v formic acid and 3% dimethylformamide [DMSO] v/v in water) and solvent B (0.2% v/v formic acid and 3% DMSO in ethanol) at a flow rate of 3 μL·min^−1^.

### *In silico choxAB* sequence analysis

The nucleotide sequence obtained for *choxAB* and its translated protein amino acid sequence (obtained using Expasy, Swiss Bioinformatics Resource Portal, https://web.expasy.org/translate/) were searched against the International Nucleotide Sequence Database Collaboration (INSCD) using the BLASTN online algorithm (https://blast.ncbi.nlm.nih.gov/Blast.cgi?LINK_LOC=blasthome&PAGE_TYPE=BlastSearch&PROGRAM=blastn) provided by the National Center for Biotechnology Information (NCBI). The secondary structure of the translated choxAB protein was predicted using the Pro‐origami server (http://munk.csse.unimelb.edu.au/pro‐origami), and the presence of a signal peptide was determined using neural network (NN) and Hidden Markov models built into the SignalP‐5.0 server (http://www.cbs.dtu.dk/services/SignalP/). The multiple sequence alignment of the choxAB amino acid sequence and those of cholesterol oxidases from other species were performed using clc sequence viewer 8.0 and a phylogenetic tree built to depict the evolutionary relationships of aligned sequences. The three‐dimensional (3D) choxAB protein structure was predicted using the online Local Meta‐Threading Server (LOMETS) (https://zhanglab.ccmb.med.umich.edu/LOMETS/), which uses ten threading programs, normalizes scores, and selects the top predicted 3D structures with *Z*‐score ≥ 1 to ensure the quality of the template used to build the structure. The 3D modeled structure of choxAB, taken from I‐TASSER, was refined using the 3D ^refine^ server (http://sysbio.rnet.missouri.edu/3Drefine/). The 3D ^refne^ webtool carries out reiterated structure defects and structural relaxation through molecular dynamic simulation. After that, the validity of the refined model was checked by three programs PROCHECK, VERIFY_3D, and ERRAT incorporated in the saves v.6.0 software localized at the online server (https://saves.mbi.ucla.edu/). ProSA‐web (https://prosa.services.came.sbg.ac.at/prosa.php) was used to determine the *z*‐score that would show the overall quality of the model. The superimposition of the 3D structures of choxAB and the most relevant PDB template was performed by pymol version 2.4 (Schrӧdinger, Inc., New York City, NJ, USA).

The theoretical pI, molecular mass, and amino acid composition of choxAB were deduced using the webtool ProtParam (https://web.expasy.org/protparam/). The presence of any transmembrane helices in the protein was predicted by the three programs SOSUI (http://harrier.nagahama‐i‐bio.ac.jp/sosui/cgi‐bin/adv_sosui.cgi), TMHMM2.0 (http://www.cbs.dtu.dk/services/TMHMM/), and PHOBIUS (https://www.ebi.ac.uk/Tools/pfa/phobius/). The motif presence was checked with Motif Scan (https://myhits.isb‐sib.ch/cgi‐bin/motif_scan).

### Cholesterol oxidase activity

Cholesterol oxidase activity assays were performed on crude soluble cell lysates from *E. coli* BL21 strain (DE3) Rosetta cells harboring the pET‐28a(+)/*choxAB* recombinant construct and induced to express choxAB according to the previously reported Richmond assay [21]. Briefly, 3.0 mL phosphate buffer (100 mm, pH 7.0) including 0.5 mL Triton X‐100/L, at 30 °C, 50 μL of enzyme preparation, and 50 μL of cholesterol in isopropanol (6.0 mm) were added in a cuvette of 10 mm light path. The blank was buffered Triton X‐100. The change in absorbance was measured at 240 nm. One unit of cholesterol oxidase activity equals the amount of enzyme that liberates 1 µmole of oxidized cholesterol under the assay conditions.

### Capillary isoelectric‐focusing analyses

The isoelectric point (pI) of recombinant expressed choxAB was determined using the whole capillary‐imaged capillary isoelectric‐focusing **(**cIEF) system (model iCE3) (ProteinSimple, Canada) according to a previously published procedure [22]. The used pI markers throughout the present work had pI values of 6.61 (102409) and 9.22 (102231). Moreover, the pI marker values 5.12 (102224), 6.14 (102220), 7.05 (102226), 8.18 (102408), and 9.22 (102231) were used to establish the standard curve. All pI markers were obtained from ProteinSimple. The crude lysate of inclusion bodies ran on 10% SDS/PAGE. After the termination of electrophoresis, the band of 62 kDa (recombinant expressed choxAB) was excised and eluted from the gel. Then, the eluted choxAB was subjected to the protocol of Gervais and king to be processed through the capillary‐imaged cIEF system (model iCE3) to determine the pI of choxAB.

## Results

### *Acinetobacter* sp. strain RAMD sequence identification

**Fig. 1:**
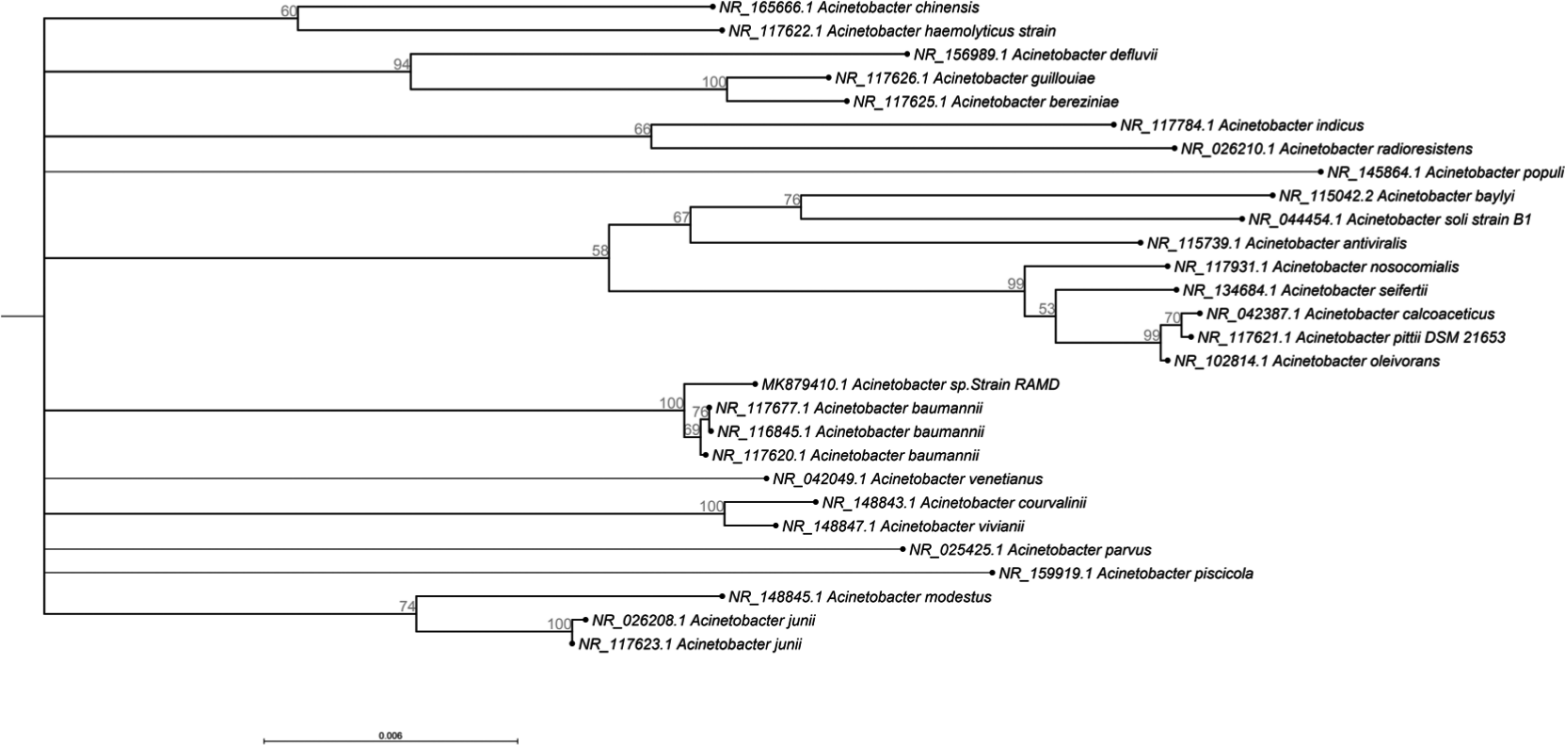
Neighbor‐joining phylogenetic tree depicting the relatedness of 16S rDNA nucleotide sequences among *Acinetobacter* sp. strain RAMD and other selected bacterial species. Bootstrap values (500 resamplings) are represented on the branch nodes. The tree was built by clc sequence viewer 8.0.

Data derived from MALDI‐TOF‐MS assigned the clinical isolate to the genus *Acinetobacter* with a score of 2.1, which is a solid identification on the genus level. Furthermore, the BLASTN search of the 16S rDNA sequence assigned the isolate to *Acinetobacter baumannii* with 99% identity and 100% query coverage. Despite the high discriminatory power of 16S rRNA sequencing for the identification of other bacterial genera, it is not powerful to differentiate *Acinetobacter* at the species level [23]. Consequently, the 16S rDNA sequence of the clinical isolate was deposited in GenBank under the accession number MK879410.1, with the designation of *Acinetobacter* sp. strain RAMD. A phylogenetic tree depicting the evolutionary relationship of *Acinetobacter* sp. strain RAMD to other related bacterial species is given in Fig. 1.

### Sequence analysis and phylogenetic relationships of *choxAB*

**Fig. 2:**
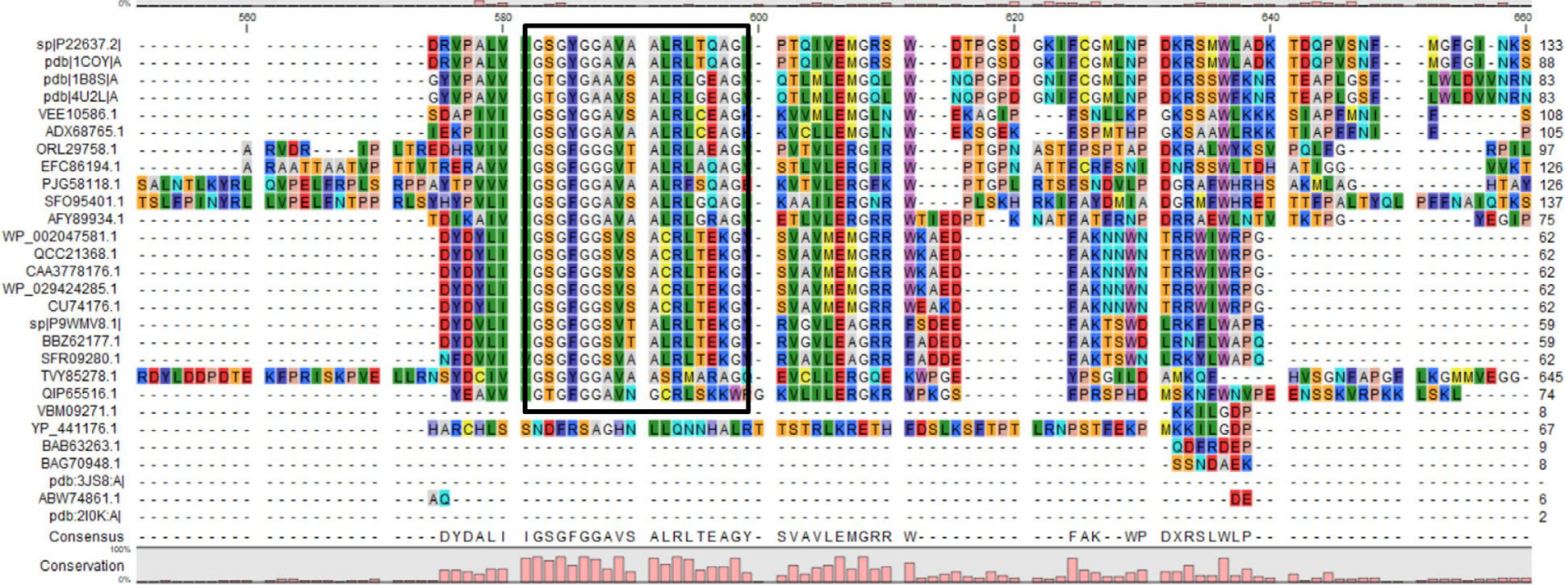
Multiple amino acid sequence alignment of choxAB and selected cholesterol oxidases from PDB and the nonredundant NCBI database. The cholesterol oxidases with the following accession numbers SplP22637.2, pdbl1COYlA, pdbl188SlA, pdbl4UZLlA, VFF10586.1, AOX68765.1, ORL29758.1, EFC86194.1, PJG58118.1, SFO95401.1, AFY89934.1, WP_002047581.1, QCC21368.1, CAA3778176.1, WP_029424285.1, CU74176.1, SPlP9WMV8.1, BBZ62177.1, SPR09280.1, TVY85278.1, QIP65516.1, VBM09271.1, YP_441176.1, BAB63263.1, BAG70948.1, pbdl3JS8lA, ABW74861.1, and pdbl210klA belong to *B. sterolicum*, *B. sterolicum*, *Streptomyces* sp., *Streptomyces* sp.SA‐COO, *C. gleum*, *Weeksella virosa*, *R. hoagii*, *Frankia* sp., *A. cavernicola*, *Enterovibrio norvegicus*, *Chroocococadiopsis thermalis*, *A. baumannii*, *Acinetobacter* sp. Strain RAMD, *A. baumannii*, *Acinetobacter* sp., *A. baumannii*, *M. tuberculosis*, *Mycolicibacterium monacensa*, *Lentzea waywayandensis*, *Lachnellula suecica*, *Leptospira interrogans*, *B. pseudomallei*, *B. thailandensis*, *B. cepacia, Chromobacterium* sp., *Chromobacterium* sp.DS‐1, *R. erytthropolsis*, and *B. sterolicum*, respectively. The rectangle indicated the conserved FAD‐binding sequence (GSGFGGSVSACRLTEKG) of class I cholesterol oxidases. The other remaining amino acid sequences of cholesterol oxidases, outside the rectangle, belonged to class II cholesterol oxidases.

**Fig. 3:**
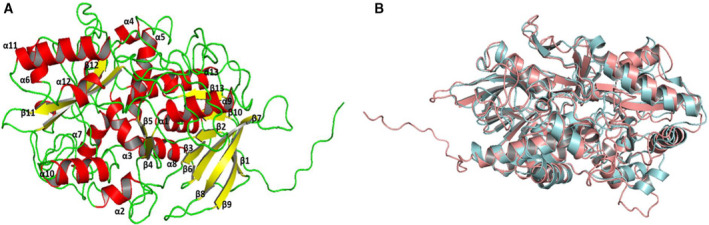
The refined 3D predicted model of choxAB in a cartoon view. (A) Refined 3D predicted model of choxAB by the online program 3D ^refine^, showed 13 α‐helices and 13 β‐sheets. (B) Superimposed cartoon view of the backbones of choxAB (cyan) and 2gewA (faint red).

The full‐length cholesterol oxidase ORF from *Acinetobacter* sp. strain RAMD was cloned, consisting of 1671 bp (Fig. S1), with 556 deduced amino acid residues; the cloned gene was designated *choxAB*, and its nucleotide sequence was deposited in GenBank under the accession number MK575469.2. A BLASTN sequence similarity search revealed that the obtained *choxAB* nucleotide sequence matched with 99.04% identity with the locus_tag="ATCC19606_26070 from *Acinetobacter baumannii* ATCC19606 DNA (GenBank: AP022836.1) with the product cholesterol oxidase of protein ID: BCB00272.1. A BLASTp sequence similarity search confirmed that the deduced amino acid sequence of choxAB had high homology to the glucose–methanol–choline (GMC) family oxidoreductase (99.82%, NCBI: WP_002047581.1) and *Acinetobacter baumannii* cholesterol oxidase (99.46%, gb: HCU74176.1). Conversely, choxAB showed low homology with cholesterol oxidases from the following species: *M*.* tuberculosis* (45.83% identity, UniProtKB: P9WMV8.1), *Brevibacterium sterolicum* (25.04% identity, PDB: 1coyA), and *Streptomyces* sp. SA‐COO (27.27% identity, PDB: 2GEW). In terms of sequence features, the choxAB amino acid sequence was found to lack a signal peptide according to the analysis performed through the Signal IP server. Meanwhile, the conserved FAD‐binding sequence (G17xG19xG21xxxxA26xxxxxxG followed by E40) [24, 25] was present in the N‐terminal of choxAB as G14SG16FG18GSVSA23CRLTE28KG followed by E37 (Fig. S2), spanning from the 14^th^ to 30^th^ amino acids. The multiple amino acid sequence alignment of choxAB and selected cholesterol oxidases from PDB and the nonredundant NCBI database revealed that choxAB is more similar to class I cholesterol oxidases (Fig. 2), in which FAD is not covalently bound. A comparison of the aligned choxAB and the most relevant PDB hits (1coyA and 2GEW) revealed its putative catalytic triad: E^220^, H^380^, and N^514^. The secondary structure predicted by Pro‐origami had an α/β barrel shape (Fig. S3) with 18 α‐helices and 12 β‐strands; however, the three‐dimensional structure predicted by LOMETS using as template *Streptomyces* sp. SA‐COO cholesterol oxidase (PDB: 2GEW) contained 13 α‐helices and 13 β‐strands (Fig. S4). The superimposition of the 3D structures of choxAB and 2GEW, depicted in Fig. S5, revealed their similarity. The root mean square deviation (RMSD) of the superimposed structures was 1.75. The normalized Z‐scores for the individual threading programs in LOMETS ranged from 1.97 to 11.35, indicating a good‐quality template. The refined model (Fig. 3A) of the initial 3D modeled structure of choxAB was evaluated through four estimates by the following online programs: PROCHECK, Verify 3D, ERRAT, and PROSA. Based on the data derived from the initial and refined Ramachandran plot, in the initial 3D model, 399 (83.6%) of residues were found to be in the most favored, 53 (11.1%) in the additional allowed, 16 (3.4%) in the generously allowed regions, and 9 (1.9%) in the disallowed region (Fig. S6A). In the refined model, 397 (83.2%) were grouped in the most favored, 55 (11.5%) in the additional allowed, 18 (3.8%) in the generously allowed regions, and 7 (1.5%) in the disallowed region (Fig. S6B). An analysis of the initial 3D modeled structure by Verify 3D indicated that 77.7% of the residues had scores ≥ 0.2 in the 3D/1D score (Fig. S7A), while in the refined model, 80.04% of the residues showed scores ≥ 0.2 in the 3D/1D score (Fig. S7B). ERRAT showed that the overall quality factor for the initial and refined models was 31.144 and 53.565, respectively (Fig. S8). The proSA‐web could not estimate the z‐score for the initial model. However, the z‐score for the refined model was −6.14 (Fig. S9A). The z‐score of the refined model showed that it was within the same range of *z*‐scores of other experimentally determined protein chains of similar size in the current PDB. This would, in turn, reflect the reliability of the structure. The energy plot displays the quality of the model by plotting energies in relation to the sequence position of amino acid. At most, positive values indicate the problematic part of a model. A comparable energy plot for the refined model and the template structures was presented in Fig. S9B,C.

The superimposition of the 3D structures of the refined choxAB model and 2GEW, depicted in Fig. 3B, revealed their similarity. The root mean square deviation (RMSD) of the superimposed structures was 1.81 (279 atoms to 279 atoms), likewise indicating a good similarity.

The theoretical molecular weight and pI of choxAB were estimated to be 62.055 kDa and 8.77, respectively. Similarly, the deduced amino acid composition showed high content of hydrophobic amino acids: G (9.5%), A (7.9%), V (6.3%), L (5.9%), F (5.4%), I (4.9%), P (4.3%), and M (4.1%), for 48.3% in total. The probability of choxAB being a transmembrane protein was checked by the online program Phobis of EMBL, which identified noncytoplasmic (residues 1–365), transmembrane (residues 366–385), and cytoplasmic (residues 386–556) domains (Fig. S10).

### Expression of recombinant choxAB

The expression of recombinant choxAB was induced in *E. coli* BL21 (DE3) Rosetta cells harboring the recombinant construct pET‐28a(+)/*choxAB* using 1 mm IPTG and 18 h of incubation at room temperature. The recombinant choxAB was expressed in the cell lysate of the induced recombinant *E. coli* cells in the form of insoluble, inactive protein (inclusion bodies). No expression of choxAB cholesterol oxidase was detected in SDS/PAGE (the soluble fraction of the cell lysate under these induced conditions) (Fig. S11).

**Fig. 4:**
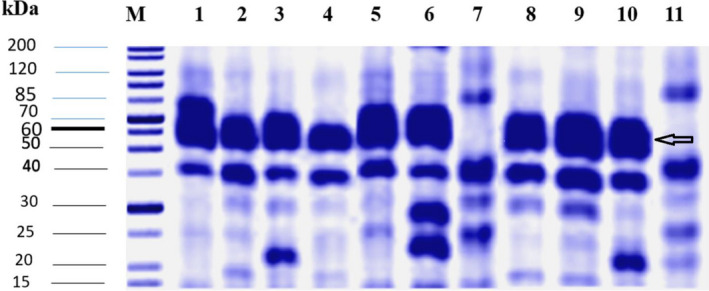
SDS/PAGE (10%) for the recombinant choxAB expressed in *E. coli* BL21 (DE3) Rosetta. Each lane contained 50 µg total protein. M: protein ladder. Lanes 1, 2, 3, 8: insoluble fractions of recombinant cells induced at room temperature and 1 mm IPTG using M9, 2xTY, 5x LB, and LB growth media, respectively. Lanes 4, 5, 6, 9, 10: insoluble fractions of recombinant cells induced at room temperature using 0.2, 0.4, 0.6, 0.8, and 1 mm IPTG, respectively. Lane 7, 11: uninduced recombinant cells.

The molecular weight of the expressed choxAB was deduced as approximately 62 kDa from SDS/PAGE (Fig. 4), consistent with *in silico* predictions.

### Optimized expression of recombinant choxAB

At most, applying the cultural conditions of using different growth media, IPTG concentrations, and incubation temperatures yielded overexpressed inactive choxAB in the insoluble fraction (Fig. 4 and Fig. S11), with a very minor level of cholesterol oxidase activity in the soluble fraction (Table S1). The induction temperature of 37 °C did not induce choxAB in the active soluble status. However, reducing the temperature did induce a bit of choxAB in the active soluble status (0.008 ± 0.00001 U·mL^−1^) at room temperature (Table S1). At low concentrations of IPTG (0.2 and 0.4 mm), no cholesterol oxidase activity of choxAB could be traced. The cholesterol oxidase activity of choxAB could be traced upon increasing the IPTG step wisely from 0.6 to 1.0 mm, with the highest activity realized at 1.0 mm IPTG (Table S1). Only an LB growth medium could support the production of choxAB with appreciable activity (0.008 ± 0.0001 U·mL^−1^) compared with other growth media (Table S1).

Reciprocally, the treatment of the choxAB inclusion bodies with the aforementioned solubilization buffers (B, C, E, and F) did succeed to solubilize the inclusion bodies, but it did not refold the expressed choxAB properly (Fig. S12), as no cholesterol oxidase activity could be recorded (Table S2). Similarly, the solubilized inclusion bodies of choxAB with the SDS did not show any cholesterol oxidase activity (Table S2).

Upon tracing the cholesterol oxidase activity in the soluble fraction of the cell lysate of recombinant *E. coli* cells, previously induced in the presence of glycerol, sorbitol, and ethanol additives, appreciable activity was recorded (Table 1 and Fig. S13). The highest appreciable cholesterol oxidase activity could be traced in the following order: 2% (v/v) glycerol >> 0.2 m sorbitol > 0.3 m sorbitol > 3% (v/v) glycerol > 0.4 m sorbitol > 4% (v/v) glycerol > 5% (v/v) glycerol > 1% (v/v) glycerol = 1% (v/v) ethanol. In contrast, no cholesterol oxidase activity could be noticed in the presence of 3% (v/v) ethanol and the three tested glucose concentrations. Impressively, using 2% (v/v) glycerol resulted in a dramatic improvement in the yield of choxAB activity from 0.008 ± 0.0001 to 0.45 ± 0.0001 U·mL^−1^, with 56.25‐fold enhancement (Table 1).

**Table 1:** Profile of recombinant choxAB cholesterol oxidase activity from cell lysate of recombinant *E. coli* cells induced in the presence of some additives.

Additive^a^	choxAB activity^b^ (U·mL^−1^)
Uninduced recombinant *E. coli* cells	0.00
Induced recombinant *E. coli* cells without any additives	0.008 ± 0.0001
Induced recombinant *E. coli* cells without any additives at 100 rpm	0.005 ± 0.0001
Induced recombinant *E. coli* cells in the presence of ethanol additive	
1%(v/v) Ethanol	0.010 ± 0.0002
2%(v/v) Ethanol	0.003 ± 0.0004
3%(v/v) Ethanol	0.00
Induced recombinant *E. coli* cells in the presence of glycerol additive	
1%(v/v) Glycerol	0.010 ± 0.0003
2%(v/v) Glycerol	0.457 ± 0.0001
3%(v/v) Glycerol	0.046 ± 0.0004
4%(v/v) Glycerol	0.020 ± 0.0005
5%(v/v) Glycerol	0.015 ± 0.0003
Induced recombinant *E. coli* cells in the presence of sorbitol additive	
0.1 m Sorbitol	0.002 ± 0.0002
0.2 m Sorbitol	0.120 ± 0.0001
0.3 m Sorbitol	0.110 ± 0.0003
0.4 m Sorbitol	0.030 ± 0.0005
Induced recombinant *E. coli* cells in the presence of glucose additive	
5 mm Glucose	0.00
10 mm Glucose	0.00
15 mm Glucose	0.00

^a^ All additives were added at the time of induction with 1 mm IPTG

^b^ Cholesterol oxidase activity was determined in the soluble fraction of recombinant *E. coli* cells according to Richmond assay as mentioned in Methods' section. All values were expressed as the average of three readings ± standard error.

### Purification of solubilized active choxAB

**Fig. 5:**
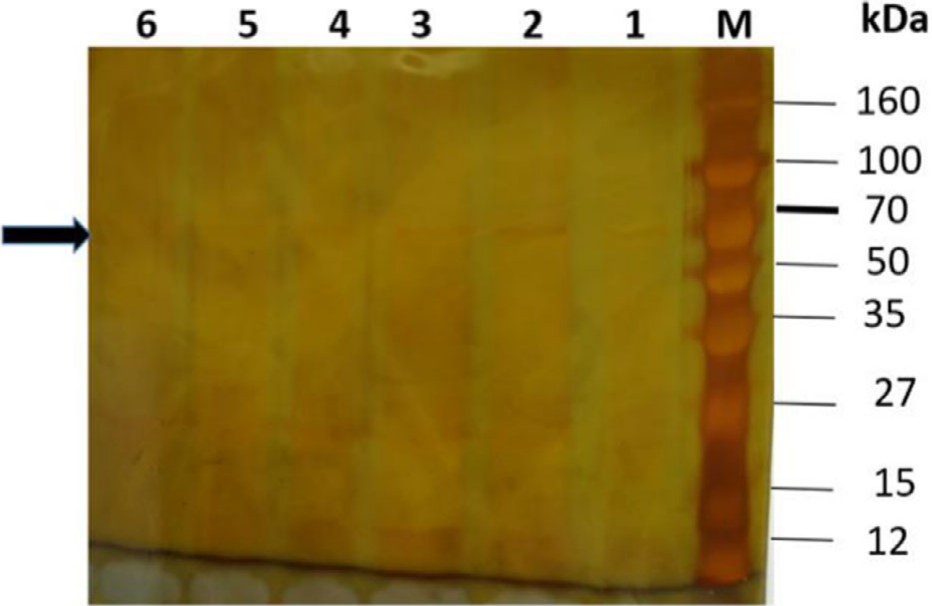
SDS/PAGE (10%) stained with silver staining for the partially purified recombinant choxAB after Ni^2+^‐NTA column through a small scale batch mode. Lanes 1–6: collected fractions after elution of the bound proteins with 500 mm Imidazole and 300 mm NaCl. Lane M: protein ladder. Arrow indicated the purified choxAB band of 62 kDa.

The data of the preliminary experiment on a small scale for the purification of the recombinant choxAB were displayed in Table 2 and Fig. 5. The choxAB recombinant enzyme was partially purified to homogeneity using a Ni^2+^‐NTA affinity matrix in a batch mode, with a specific activity, yield, and fold of 0.054 U·mg^−1^, 8.1%, and 11.69, respectively (Table 2). Despite the low yield of the partially purified to homogeneity recombinant choxAB, it was traced on SDS/PAGE (Fig. 5).

**Table 2:** Purification table of recombinant choxAB produced by *E. coli* BL21 (DE3) Rosetta.

Purification step	Total units	Total mg protein	Specific activity (U·mg^−1^)	Fold purification	Yield (%)
Crude cell lysate	0.88	190.2	0.0046	1.00	100.00
After Ni^2+^‐agarose affinity column	0.071	1.32	0.054	11.69	8.1

### Isoelectropoint of recombinant choxAB

**Fig. 6:**
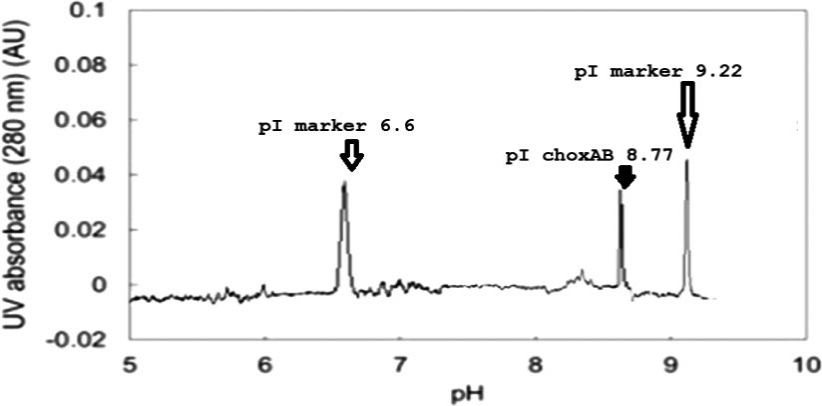
Typical experimental choxAB cIEF profile along with two standard protein pI markers. *Y*‐axis: The absorbance at 280 nm reflects the presence of a precipitated protein at a certain position in the capillary gel. The intensity of the protein peak evidences its concentration as deduced from the magnitude of its absorbance value at 280 nm. *X*‐axis: the pH at which the precipitation of the protein occurs; synonymously called isoelectric point (pI). The position of the choxAB is at pI 8.77 value (middle peak) as pointed out by the filled arrow. The positions of the two standard proteins pI marker peaks are indicated by pI values of 6.6 and 9.22 as pointed out by the empty arrows.

The laboratory data of pI for recombinant choxAB (performed with capillary isoelectric‐focusing analyses) did reveal a value of 8.77, as shown in Fig. 6.

### Identification of the recombinant expressed choxAB by LC‐MS‐MS

LC‐MS/MS confirmed that the inclusion bodies in the 62 kDa band obtained from the sonicated cell lysates of *E. coli* BL21 (DE3) Rosetta cells harboring the recombinant construct pET‐28a(+)/*choxAB* showed a 100% matched identity to *A. baumannii* cholesterol oxidase (UniProtKB: A0A0E1FG24), with an 82.6% sequence coverage (Fig. S14).

## Discussion

Despite CHOX enzymes being available from both pathogenic and nonpathogenic wild‐type bacteria, three major hurdles limit their industrialization and commercialization: extremely low productivity, multistep purification protocols, and possible hazards inherent in handling pathogenic CHOX producers. Typically, the cloning expression of CHOXs using the heterologous host *E. coli*, a species generally regarded as safe (GRAS), is considered the gold standard solution for avoiding these obstacles. In this regard, great attention has been paid in recent years toward the cloning and expression of CHOXs from a wide range of pathogenic and nonpathogenic bacteria [2, 26, 27, 28, 29, 30, 31, 32, 33], especially after the publication of a plethora of microbial genome sequences by the NCBI. Nevertheless, the cloning of CHOX‐encoding genes from their wild‐type producers is still considered a venture in its infancy. In addition, it remains necessary to continue tracing novel CHOXs to cope with the demands of worldwide enzyme markets. In this light, the current study was the first to conduct the cloning, expression, and *in silico* molecular characterization of a CHOX ORF fragment (*choxAB*) from the locally isolated clinical pathogen *Acinetobacter* sp. strain RAMD in the *E. coli* strain BL21 (DE3) Rosetta.

The successful cloning and expression of the putative ORF encoding cholesterol oxidase (gb locus tag: IX87_05230), in turn, confirm the correctness of this annotated locus, which exists in the published genomes of several strains of *A. baumannii*. Moreover, the BLASTp search of the *choxAB* translated amino acid sequence revealed its high similarity to previously reported *A. baumannii* cholesterol oxidase protein (99.4%, gb: HCU74176.1). Meanwhile, its low similarity to proteins from *B. sterolicum* (25.04%, PDB: 1coy_A) and *Streptomyces* sp. SA‐COO (27.27%, PDB: 2gew), in turn, shows the distal relation of choxAB to these CHOXs and, furthermore, the large variation in CHOX amino acid sequences among different bacteria. The total number of amino acids in choxAB (556 residues) was in good harmony with most reported cholesterol oxidases from other bacteria (500–580 amino acids), including the CHOX of *Rhodococcus* sp., *Chromobacterium* sp. DS‐1, *A. simplex*, *Burkholderia cepacia* strain ST‐200, *Streptomyces* sp. SA‐COO, *C. gleum*, and *B. sterolicum* ATCC 21387 [4].

The majority of CHOXs are reported to have an N‐terminal signal peptide (40–50 amino acids) that precedes the mature protein [4, 34]; however, an analysis of the choxAB amino acid sequence revealed the absence of a signal peptide at its N terminus. This suggests that the mode of secretion for choxAB might be either intracellular or via a membrane‐bound enzyme. In addition, class I CHOXs (noncovalent FAD‐binding) are characterized by a fingerprint consensus FAD‐binding motif with repeating glycine residues at their N terminus (G17xG19xG21xxxxA26xxxxxxG, followed by E40) [24, 25]; this is seen in the CHOX protein structures of *Streptomyces* sp. SA‐COO (PDB: 1B4V) and *B. sterolicum* (PDB: 1COY_A) [5, 6, 31, 35]. Consistent with this class, choxAB contained the conserved FAD‐binding motif G14SG16FG18GSVSA23CRLTE28KG followed by E37 at its N terminus. This conserved sequence of glycine residues mediates the binding of the charged diphosphate moiety of a nucleotide near the N‐terminal end of a helix, with stabilization imposed by the helix dipole force [36]. The presence of the noncovalent FAD‐binding consensus motif would suggest the existence of a nucleotide‐binding fold. Conversely, class II CHOXs (covalent FAD‐binding) lack this sequence of glycine residues [24], implying the probable deficiency of a nucleotide‐binding fold in those proteins. Although the class I consensus FAD‐binding glycine motif is present in choxAB, its affiliation to class I should be further supported by conducting a thorough analysis of its experimental crystal structure in a future study.

The secondary structure predicted for choxAB by the Origami server revealed the presence of 18 α‐helices and 12 β‐strands (Fig. S3). In contrast, the topology inferred from three‐dimensional structure predictions by LOMETS exhibited 16 α‐helices and 8 β‐strands (Fig. 3A). Unlike choxAB, the CHOX proteins of *B. sterolicum* (1coyA) and *Streptomyces* sp. (2GEW) both contain 12 α‐helices and 18 β‐strands. The multiple amino acid sequence alignment of choxAB and these two well‐studied experimental 3D structures identified their catalytic triads: E^220^‐H^380^‐N^514^ (choxAB), E^361^‐ H^447^‐N^485^ (1coyA), and E^356^‐H^442^‐N^480^ (2GEW).

An appreciable cholesterol oxidase activity of choxAB was realized at low induction temperatures (i.e., room temperature). Reportedly, applying reduced induction temperatures is one of the established strategies to decrease the likelihood of recombinant proteins’ formation of inclusion bodies [37]. At most, a higher induction temperature at 37 °C does encourage the hydrophobic interactions that would lead to the formation of inclusion bodies [38]. At low induction temperatures, T7‐based expression systems such as pET‐28a(+) are repressed, minimizing the yield of inclusion bodies accompanied by a noticeable increase in the yield of soluble protein [39]. Additionally, low induction temperatures improve recombinant protein solubility at the expense of its yield. Using high IPTG concentration at 1.0 mm does improve the solubility of the recombinant choxAB (0.008 ± 0.0001 U·mL^−1^) compared with other concentrations of IPTG. Our finding was in disagreement with previous findings, stating that using high concentrations of IPTG does increase the yield of recombinant protein at the expense of its solubility [16, 29]. One possible explanation of the increased solubility of choxAB at a high IPTG concentration (1.0 mm) is that the strong T7 promoter of the pET‐28+(a) requires a high IPTG concentration to turn on the gene. Generally, the optimal concentration of IPTG required for getting solubilized recombinant protein is variable from protein to another. The use of 0.2 m sorbitol does increase the choxAB solubility. This could be attributed to the inhibition of cell growth by sorbitol, which would reduce protein aggregation [40, 41]. However, applying a higher concentration of sorbitol (> 0.2 m) alleviated the solubility of choxAB dramatically. It might be attributed to the much greater inhibition of cell growth at high concentrations of sorbitol, leading to reduced protein production. Similarly, using a low concentration of glycerol (2% [v/v]) impressively increased choxAB solubility with the highest appreciable yield of choxAB activity (0.45 U·mL^−1^). However, using higher concentrations of glycerol (> 2% [v/v]) showed an adverse effect on choxAB solubility. This could be attributed to the nature of glycerol as a sugar alcohol imposing cell growth inhibition in a similar manner to that of the sugar alcohol sorbitol at high concentrations. In contrast, a little bit of choxAB solubility was realized at 2% (v/v) ethanol. One possible explanation is the inhibitory growth effect imposed by ethanol on the recombinant *E. coli* cells. Our finding regarding the effect of glucose addition on choxAB solubility was in accordance with previous results stating that applying high concentrations of glucose could lead to excessive acetate formation. Hence, the accumulated acetate would inhibit the cell growth and the recombinant protein production as well [42]. In general, using high concentrations of sugar as additives in the growth media would lead to a drop in pH below 6.0. This, in turn, would lead to a reduction in cell density [29]. Interestingly, the cell density recombinant protein production profile is diverse with respect to various carbon sources with dissimilar cellular uptake and recombinant protein production in *E. coli* [43, 44]. The inability to trace the choxAB activity in solubilized choxAB after the treatment with the aforementioned solubilization buffers (B, C, E, and F) could be attributed to the irreversible inhibitory effect of urea even after performing dialysis. Similarly, the SDS imposed irreversible inhibitory effects on the choxAB even after a dialysis.

Despite the low yield of the choxAB activity expressed by the recombinant *E. coli* cells, its optimized level (0.45 U·mL^−1^) could be comparable with other reported cholesterol oxidases expressed by recombinant *E. coli* cells. At most, the level of reported recombinant cholesterol oxidases is small in terms of absolute units (U·mL^−1^). For instance, the values of recombinant cholesterol oxidase in the cell lysate of recombinant *E. coli* from *Rhodococcu*s sp. [45], *Chromobacterium* sp. DS‐1 [30], *Rhodococcus* sp. PTCC 1633 [27], and *Streptomyces* sp. SA‐COO [29] were 4.0, 2.0, 1.0, and 0.158 U·mL^−1^, respectively. Additionally, the yield of cholesterol oxidase of *S. lavendulae* YAKB‐15 expressed in *S. albus* J1074 was 0.78 U·mL^−1^ [31].

The purification of the expressed choxAB was attempted as a preliminary study on a small scale in the batch mode. Despite the low yield of the partially purified choxAB from the Ni^2+^‐agarose affinity matrix (0.038 U·mg^−1^), it was purified to folds of 7.76. This successful trial might open the venue to purifying the recombinant choxAB on a higher scale to increase its yield in prospective studies.

Markedly, the molecular weight reported for CHOXs varies widely among bacteria, from 47 to 62 kDa [3]. For instance, the molecular weights of CHOXs from *B. sterolicum*, *Bordetella* sp., *A. simplex*, *Chromobacterium* sp. DS‐1, *Pseudomonas aeruginosa*, and *C. gleum* are 46.5, 55, 57, 58, 59, and 60 kDa, respectively [3]. A molecular weight of 62.055 kDa was obtained for choxAB, which was in accordance with the range. Additionally, the theoretical molecular weight agreed with the experimental molecular weight (˜ 62 kDa).

The experimental pI value (8.77) of the recombinant choxAB was in agreement with its theoretical pI value. Unfortunately, choxAB was overexpressed in *E. coli* as insoluble and inactive protein, namely, inclusion bodies (IBs), as determined from SDS/PAGE and SEM analyses. This necessitated the identity of the 62 kDa SDS/PAGE band containing the IBs confirmed on the protein level. LC‐MS‐MS confirmed that the recombinant protein band containing the IBs assigned to *A. baumannii* cholesterol oxidase (UniProtKB: A0A0E1FG24) had an 82.6% query coverage.

*Escherichia coli* is a potential microbial cell factory for the large‐scale production of recombinant proteins. However, the expression of recombinant proteins in an insoluble and inactive form, namely, inclusion bodies, is a significant hurdle and would prevent the practical use of the bioprocess [46, 47]. Such inclusion bodies result from the improper folding (misfolding) of the expressed protein. A key determinant of this misfolding may be the hydrophobic content of the expressed protein, which may promote high aggregation propensity [35, 36]. choxAB showed high aggregation propensity and high content of hydrophobic amino acids (48.3% in total). Notably, while IBs are an unwanted end product in terms of protein activity, interest in them is growing in biomedical fields due to their mechanical stability [48].

## Conclusions

This is the first report describing the cloning, expression, and *in silico* analysis of the gene cholesterol oxidase (class I, choxAB) from the pathogen *Acinetobacter* sp. strain RAMD. Despite the overexpression of the recombinant choxAB in the form of inclusion bodies, an appreciable level of choxAB activity (0.45 U·mL^−1^) was realized under optimized cultural conditions with 56.25‐fold enhancement. In addition, the recombinant choxAB was purified partially to homogeneity in a pilot experiment with a specific activity and fold purification of 0.038 U·mg^−1^ and 7.76, respectively. More strategies for decreasing the inclusion bodies such as fusion of choxAB to solubilization tags and switching to the heterologous expression host *Pichia pastoris* should be tested in prospective studies. Moreover, the characteristics of the choxAB should be investigated in future studies.

## Conflict of interest

The authors declare no conflict of interest.

## Author contributions

AME and HEM conceptualized the study. AME and HEM contributed to methodology. AME provided software. HEM and SWE validated the study. AME and SWE investigated the study. AME and HEM curated the data. AME, HEM, and SWE provided resources. AME, HEM, and SWE involved in formal analysis. AME wrote—original draft preparation. AME, HEM, and SWE wrote—review. AME and SWE edited the manuscript. AME, HEM, and SWE visualized the study. AME supervised the study.

## Supporting information

**Fig. S1**. 1% agarose electrophoresis showing the PCR product of the amplified ORF of cholesterol oxidase gene from *Acinetobacter* sp. Strain RAMD. M: DNA ladder. Lane 1: Amplified PCR fragment (1671 bp) of ORF from *Acientobacter* sp. Strain RAMD encoding the cholesterol oxidase gene.

**Fig. S2**. The primary amino acid sequence (556 amino acids) of the recombinant choxAB protein inferred from the translated open reading frame (1671 bp) of *choxAB*. Arrow indicates the start codon (M^1^); squares indicate the catalytic triad (E^220^, H^380^, and N^514^); star indicates the stop codon (TAA); rectangle indicates the conserved FAD‐binding sequence (GSGFGGSVSACRLTEKG).

**Fig. S3**. Predicted cartoon secondary structure of the translated choxAB amino acid sequence generated by Pro‐origami. β‐strands and α‐helices are indicated as arrows and cylinders, respectively, with lengths relative to the number of amino acid residues they encompass. Numbers refer to the order in which features appear in the primary protein sequence; N and C indicate the N‐ and C‐terminus of the protein, respectively.

**Fig. S4**. The initial 3D predicted model of choxAB, in a cartoon view, obtained by homology modeling by i‐TASSER LOMETS web tool.

**Fig. S5**. Superimposition, in a cartoon view, of the initial 3D predicted model of choxAB (in cyan) with the PDB template 2GEW (in faint red) of cholesterol oxidase from Streptomyces sp. SA‐COO. The RMSD value was 1.75. Superimposition was performed by pymol version 2.4.

**Fig. S6**. Ramachandran plot generated by PROCHECK the 3D predicted model of choxAB. A: the initial predicted 3D model. B: the refined 3D model of choxAB.

**Fig. S7**. Verify 3D for the predicted 3D model of choxAB. A: initial predicted 3D model of choxAB. B: refined predicted 3D model of choxAB.

**Fig. S8**. ERRAT graph for the predicted 3D model of choxAB. A & B: initial predicted 3D model of choxAB. C & D: refined predicted 3D model of choxAB.

**Fig. S9**. The ProSA‐web z‐score plot for the refined 3D model of choxAB. A: refined 3D model of choxAB. B: refined 3D model ribbon view of choxAB with lowest energy regions (blue color) and highest energy regions (red color). C: energy plot for the refined 3D model.

**Fig. S10**. Phobis posterior probability for choxAB amino acid sequence. The non‐cytoplasmic domain spans from residues 1 to 365; the transmembrane domain from 366 to 385; and the cytoplasmic domain from 386 to 556.

**Fig. S11**. SDS‐PAGE (10%) for the recombinant choxAB expressed in *E. coli* BL21 (DE3) Rosetta. Each lane contained 50 µg total protein. M: protein ladder. Lanes 1–4: soluble fractions of cell lysate of recombinant cells induced at room temperature and 1 mM IPTG using M9, 2xTY, 5x LB, and LB growth media, respectively. Lanes 5–9: soluble fractions of cell lysate of recombinant cells induced at room temperature using 0.2, 0.4, 0.6, 0.8, and 1 mM IPTG, respectively. Lanes 10–12: soluble fractions of cell lysate of recombinant cells induced using 1mM IPTG at room temperature, 30 ^o^C, and 37 ^o^C, respectively. Lane 13: uninduced soluble fraction of cell lysate of recombinant cells.

**Fig. S12**. SDS‐PAGE (10%) for the recombinant choxAB expressed in *E. coli* BL21 (DE3) Rosetta. Each lane contained 50 µg total protein. M: protein ladder. Lanes (1–4): recombinant choxAB after solubilization with four solubilizing buffers namely C, F, E, and B, respectively.

**Fig. S13**. SDS‐PAGE (10%) for the recombinant choxAB expressed in *E. coli* BL21 (DE3) Rosetta where induction (with 1 mM IPTG) was performed in the presence of the following additives at the time of induction glycerol, ethanol, sorbitol, and glucose. Panel (A): lanes 1&2: soluble fractions of cell lysate of recombinant *E.coli* cells induced in the presence of 1% (v/v) ethanol and 3% (v/v) glycerol, respectively. Lane 3: soluble fraction of cell lysate of recombinant *E.coli* cells induced without any additives. Lane 4: Protein ladder. Lane 5 & 7: soluble fractions of cell lysate of recombinant *E.coli* cells induced in the presence of 0.2 M sorbitol and 15 mM glucose, respectively. Lanes 6 & 10: soluble fractions of cell lysate of uninduced recombinant *E.coli* cells. Lane 8 & 9: insoluble fractions of cell lysate of recombinant *E.coli* cells induced in the presence of 1% (v/v) ethanol and 0.2 M sorbitol, respectively. Panel (B): lanes 1 & 5 soluble fractions of cell lysate of uninduced recombinant *E.coli* cells, lane 2: insoluble fraction of cell lysate of recombinant *E.coli* cells induced in the presence of 0.3 M sorbitol, lane 3: soluble fraction of cell lysate of recombinant *E.coli* cells induced without any additives, lane 4: Protein ladder. Lanes 6–10: soluble fractions of cell lysate of recombinant *E.coli* cells induced in the presence of 5% (v/v) glycerol, 4% (v/v) glycerol, 3% (v/v) glycerol, 2% (v/v) glycerol, and 1% (v/v) glycerol, respectively. Panel (C): lanes 1 & 10: insoluble fractions of cell lysate of recombinant *E.coli* cells induced in the presence of 3% (v/v) glycerol and 4% (v/v) glycerol, respectively. Lanes 2 & 7: soluble fractions of cell lysate of uninduced recombinant *E.coli* cells, lanes 3–6: soluble fractions of cell lysate of recombinant *E.coli* cells induced in the presence of 0.4 M sorbitol, 0.3 M sorbitol, 0.2 M sorbitol, and 0.1 M sorbitol, respectively. Lane 8: Protein ladder. Lane 9: soluble fraction of cell lysate of recombinant *E.coli* cells induced without any additives. Panel (D): lane 1: Protein ladder. Lanes 2–7: soluble fractions of cell lysate of recombinant *E.coli* cells induced in the presence of 15 mM glucose, 10 mM glucose, 5 mM glucose, 3%(v/v) ethanol, 2%(v/v) ethanol, and 1%(v/v) ethanol, respectively. Lane 8: soluble fraction of cell lysate of uninduced recombinant *E.coli* cells, lane 9: soluble fraction of cell lysate of recombinant *E.coli* cells induced without any additives. Lane 10: insoluble fraction of cell lysate of recombinant *E.coli* cells induced in the presence of 2% (v/v) glycerol.

**Fig. S14**. Concordance of choxAB amino acid sequence obtained via tryptic digestion and LC‐MS‐MS with that of *Acinetobacter baumannii* cholesterol oxidase (UniprotKB: A0A0E1FG24, 82.6% sequence coverage). Confidence of choxAB amino acids is indicated by color: green, good; yellow, mid; and red, low confidence. Gray letters represent amino acids not seen in a peptide of choxAB profiled by LC‐MS‐MS.

**Table S1**. Levels of cholesterol oxidase activity of choxAB from the cell lysate of induced recombinant *E.coli* cells under different cultural conditions.

**Table S2**. Levels of solubilized cholesterol oxidase activity of choxAB in the insoluble fraction of the cell lysate of recombinant *E.coli* cells.

## Data Availability

All data generated or analyzed during this study are included in this published article.
