# Supplementary figures and images for: Cloning, expression, and in silico structural modeling of cholesterol oxidase of Acinetobacter sp. strain RAMD in E. coli
Source: FEBS Open Bio. 2021 Jul 31;11(9):2560–75. doi: 10.1002/2211-5463.13254 (PMC8409315; doi:10.1002/2211-5463.13254)

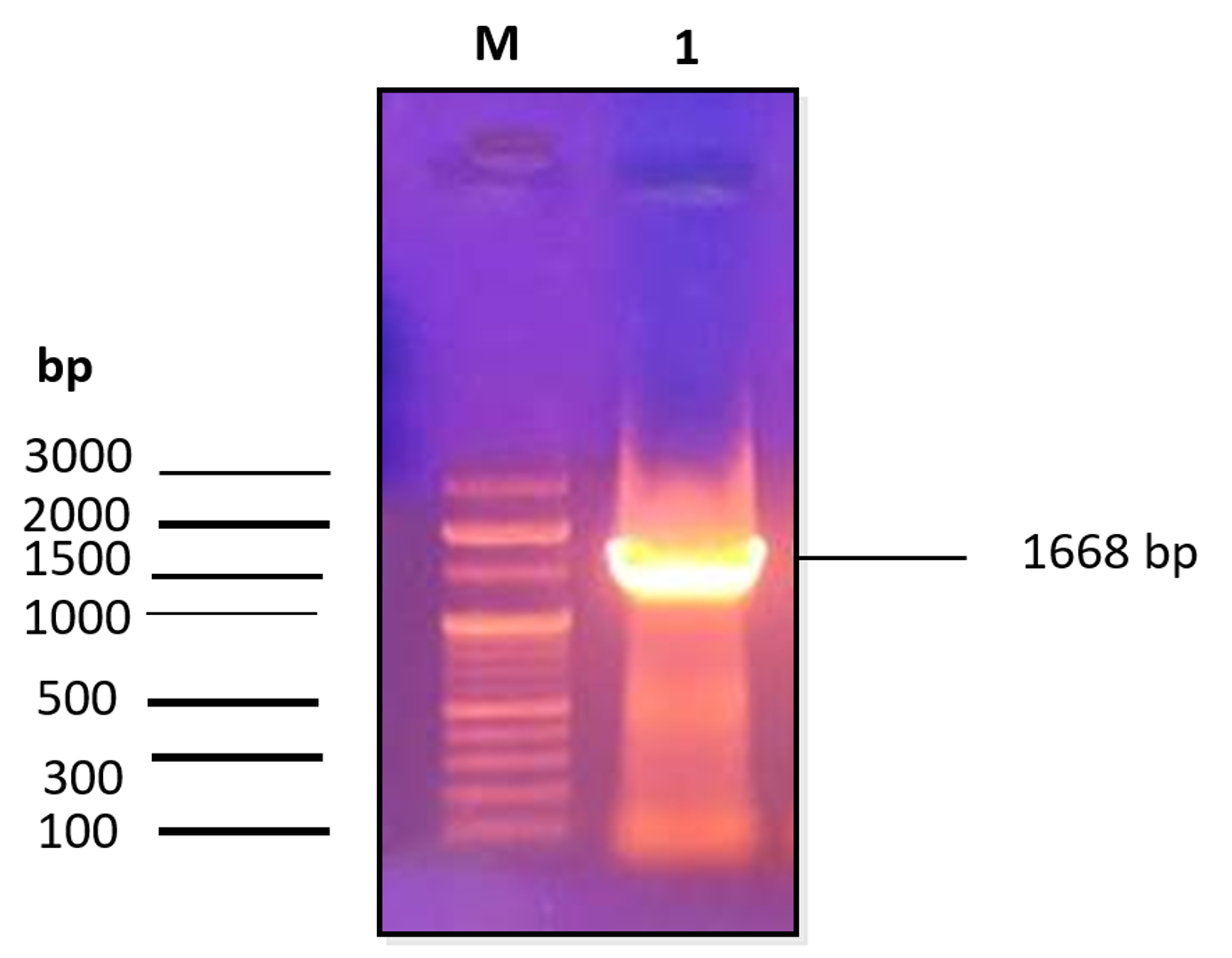

Supplement: Supplementary file 1 — Fig. S1. 1% agarose electrophoresis showing the PCR product of the amplified ORF of cholesterol oxidase gene from Acinetobacter sp. Strain RAMD. M: DNA ladder. Lane 1: Amplified PCR fragment (1671 bp) of ORF from Acientobacter sp. Strain RAMD encoding the cholesterol oxidase gene. [file FEB4-11-2560-s015.tif]

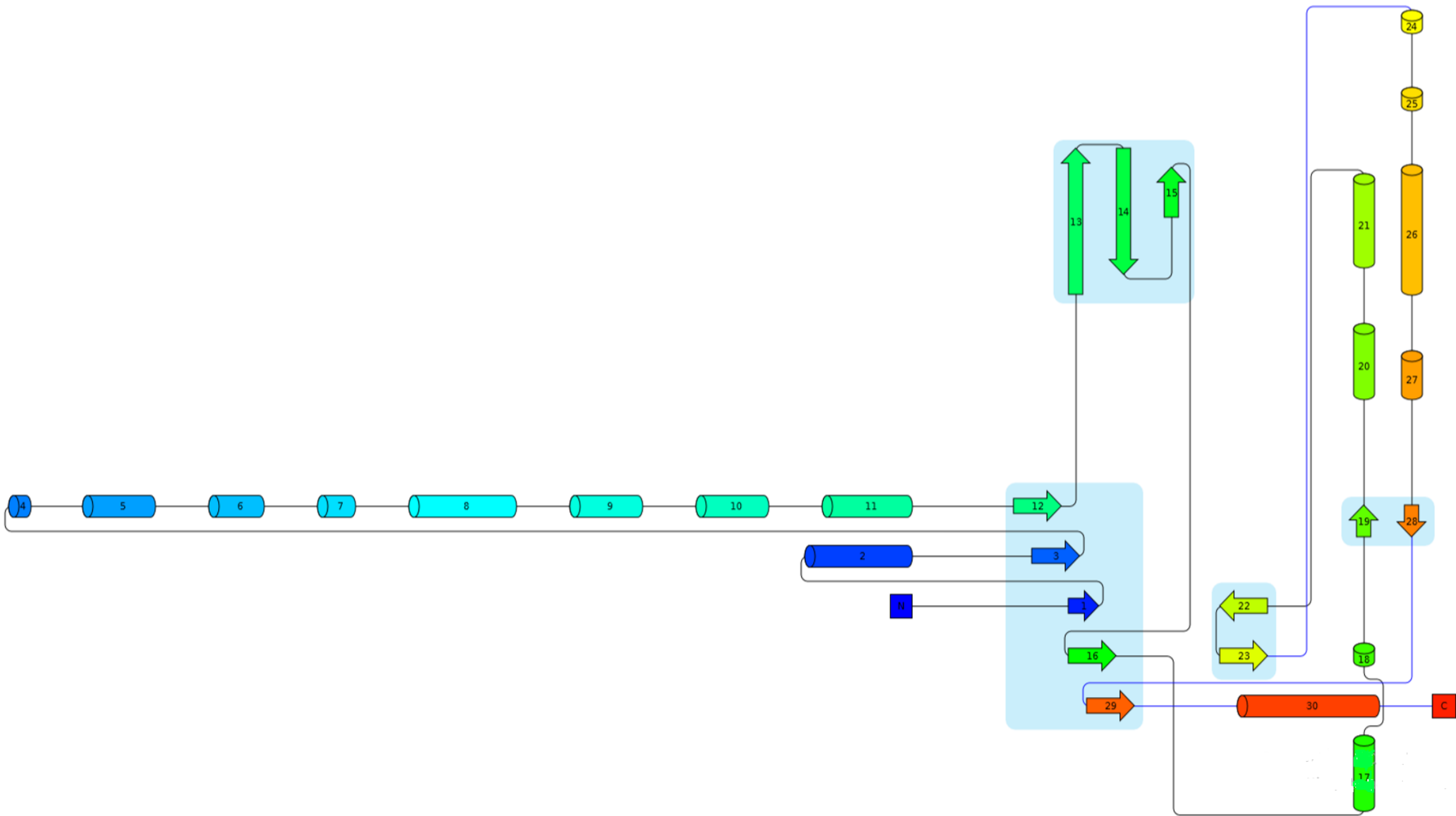

Supplement: Supplementary file 3 — Fig. S3. Predicted cartoon secondary structure of the translated choxAB amino acid sequence generated by Pro‐origami. β‐strands and α‐helices are indicated as arrows and cylinders, respectively, with lengths relative to the number of amino acid residues they encompass. Numbers refer to the order in which features appear in the primary protein sequence; N and C indicate the N‐ and C‐terminus of the protein, respectively. [file FEB4-11-2560-s014.pdf]

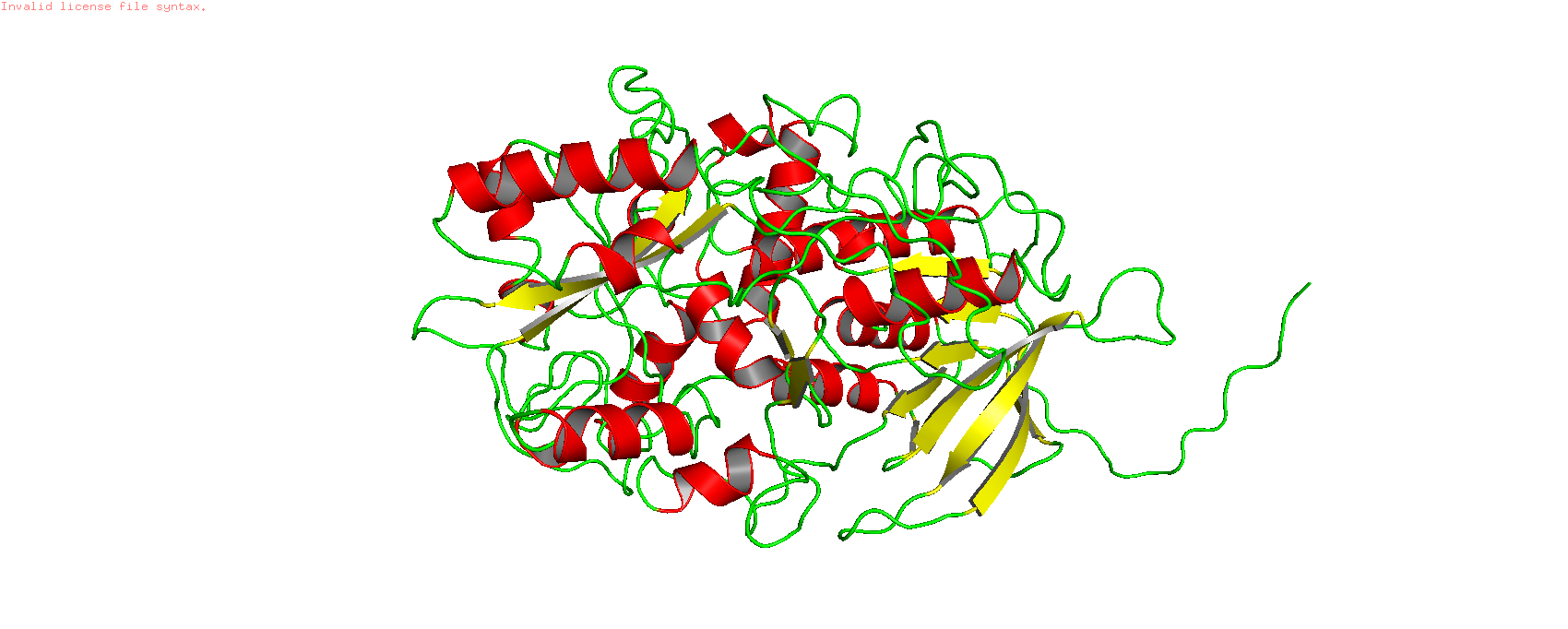

Supplement: Supplementary file 4 — Fig. S4. The initial 3D predicted model of choxAB, in a cartoon view, obtained by homology modeling by i‐TASSER LOMETS web tool. [file FEB4-11-2560-s006.tif]

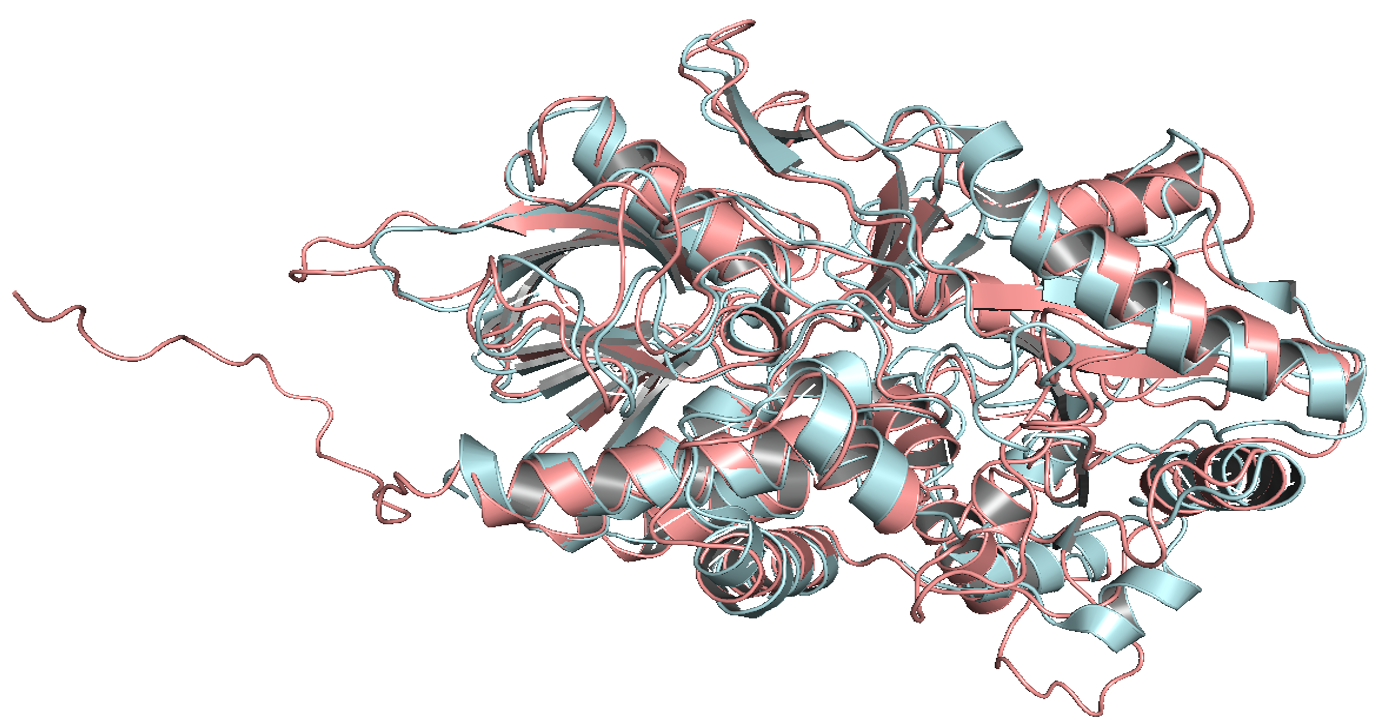

Supplement: Supplementary file 5 — Fig. S5. Superimposition, in a cartoon view, of the initial 3D predicted model of choxAB (in cyan) with the PDB template 2GEW (in faint red) of cholesterol oxidase from Streptomyces sp. SA‐COO. The RMSD value was 1.75. Superimposition was performed by pymol version 2.4. [file FEB4-11-2560-s008.tif]

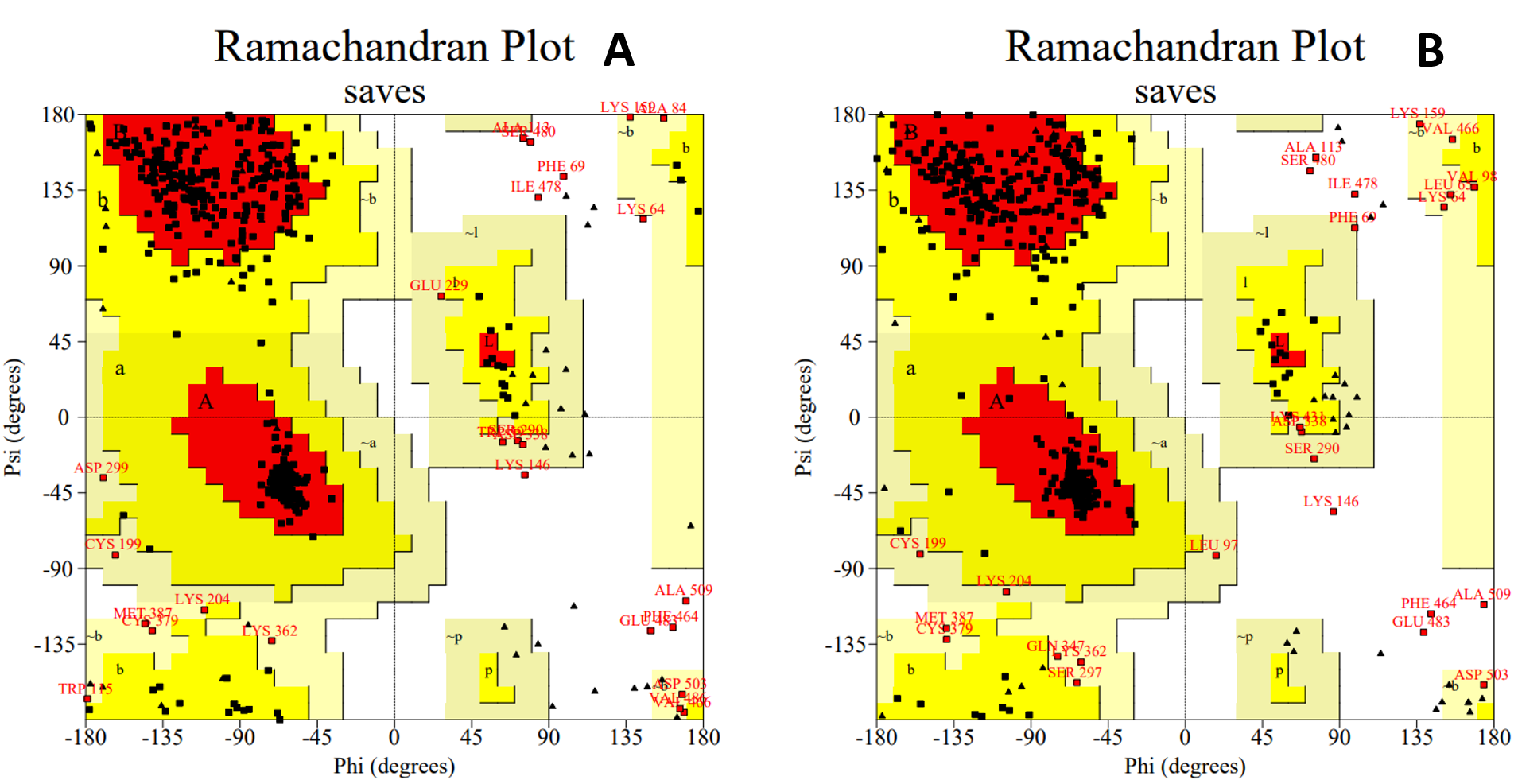

Supplement: Supplementary file 6 — Fig. S6. Ramachandran plot generated by PROCHECK the 3D predicted model of choxAB. A: the initial predicted 3D model. B: the refined 3D model of choxAB. [file FEB4-11-2560-s004.tif]

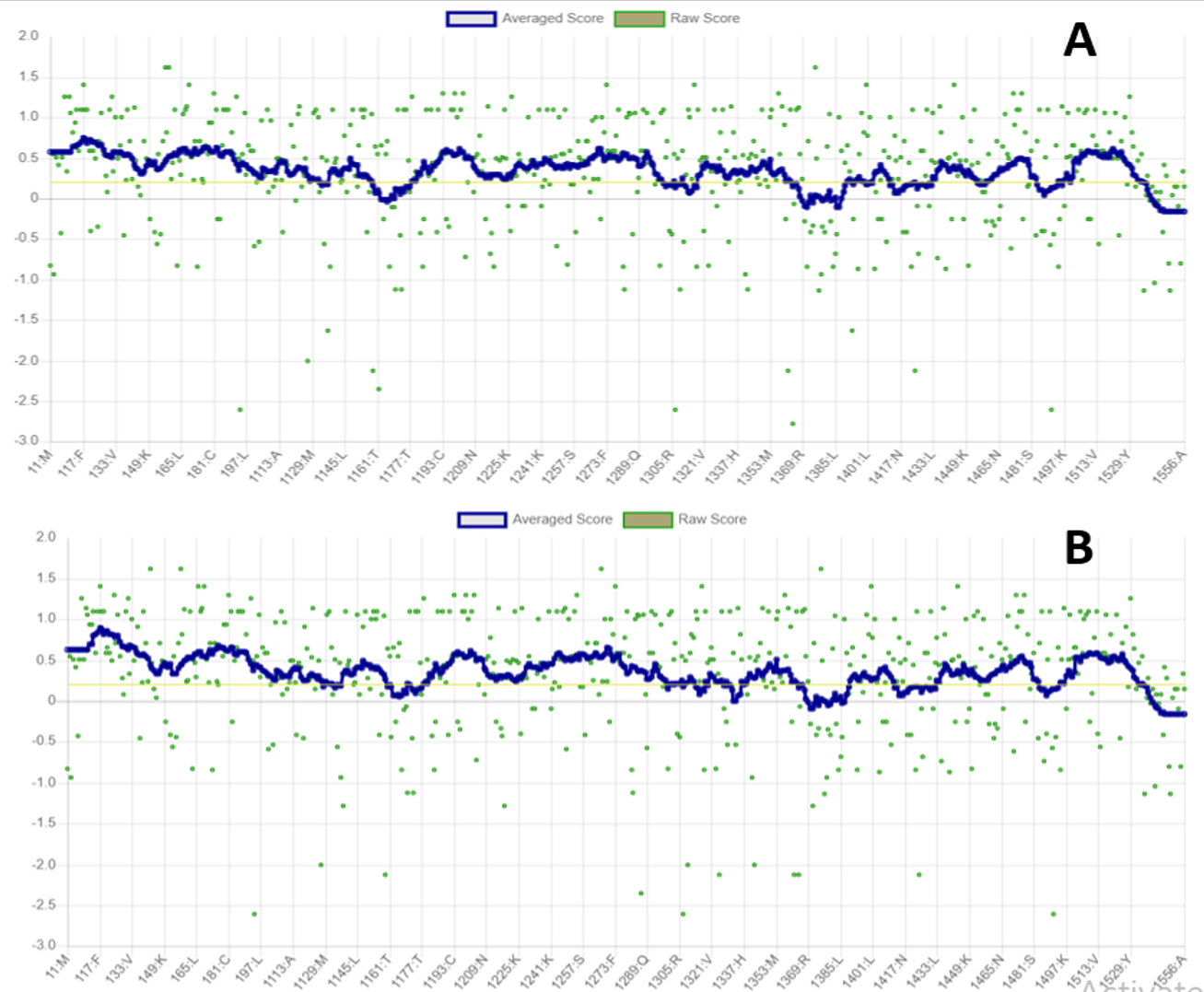

Supplement: Supplementary file 7 — Fig. S7. Verify 3D for the predicted 3D model of choxAB. A: initial predicted 3D model of choxAB. B: refined predicted 3D model of choxAB. [file FEB4-11-2560-s009.tif]

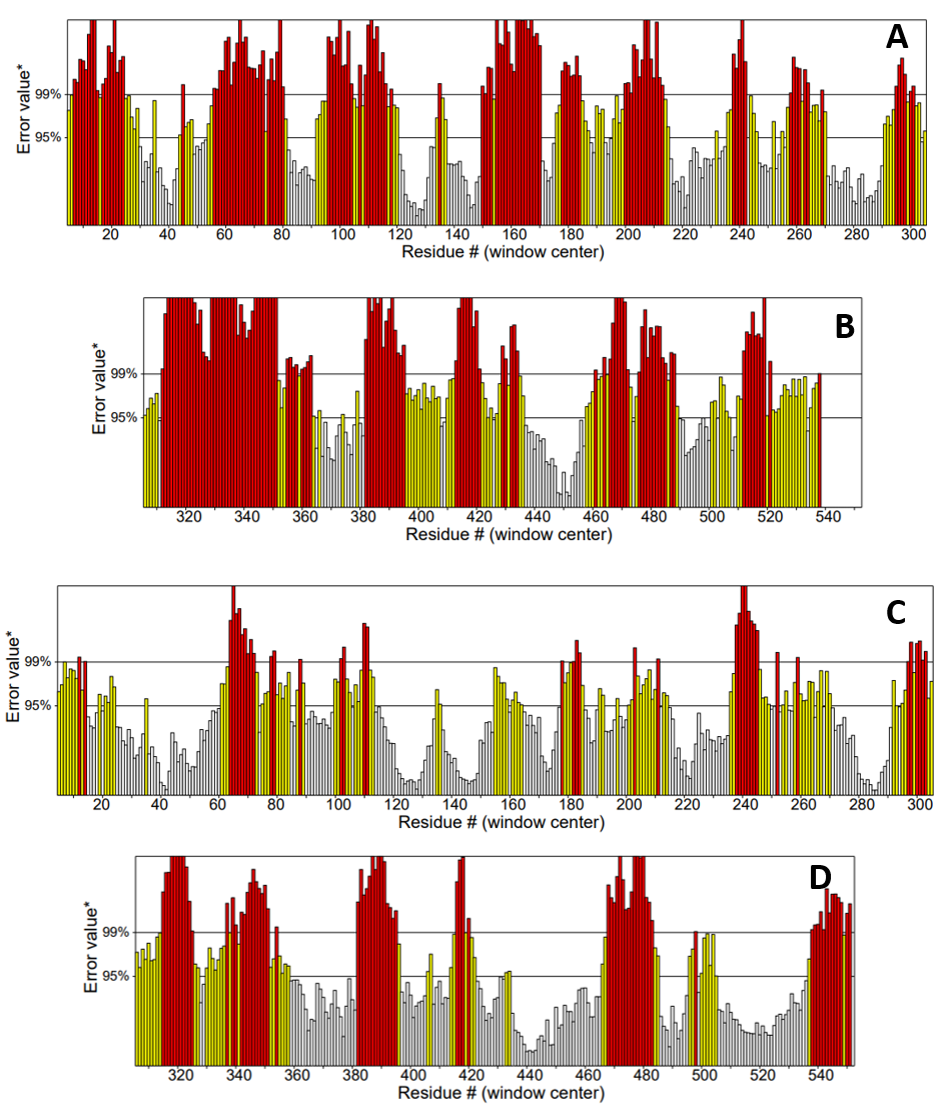

Supplement: Supplementary file 8 — Fig. S8. ERRAT graph for the predicted 3D model of choxAB. A & B: initial predicted 3D model of choxAB. C & D: refined predicted 3D model of choxAB. [file FEB4-11-2560-s011.tif]

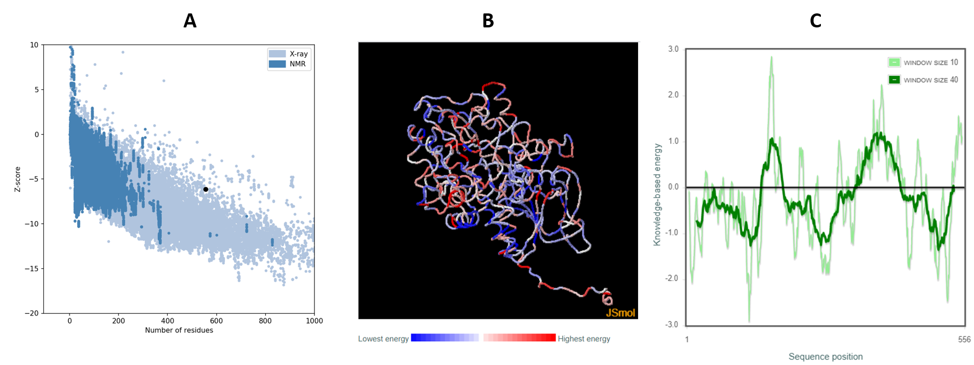

Supplement: Supplementary file 9 — Fig. S9. The ProSA‐web z‐score plot for the refined 3D model of choxAB. A: refined 3D model of choxAB. B: refined 3D model ribbon view of choxAB with lowest energy regions (blue color) and highest energy regions (red color). C: energy plot for the refined 3D model. [file FEB4-11-2560-s013.tif]

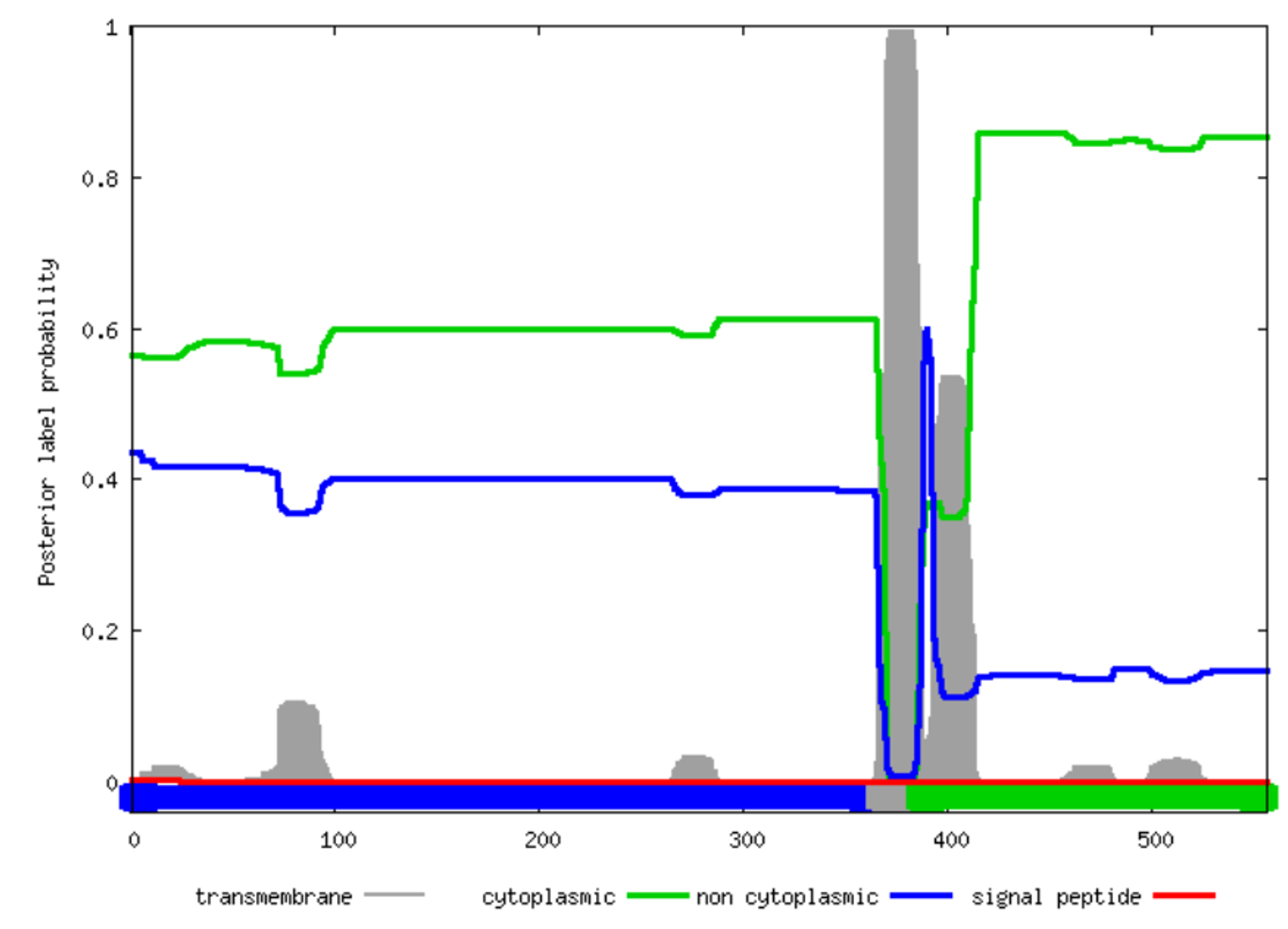

Supplement: Supplementary file 10 — Fig. S10. Phobis posterior probability for choxAB amino acid sequence. The non‐cytoplasmic domain spans from residues 1 to 365; the transmembrane domain from 366 to 385; and the cytoplasmic domain from 386 to 556. [file FEB4-11-2560-s005.tif]

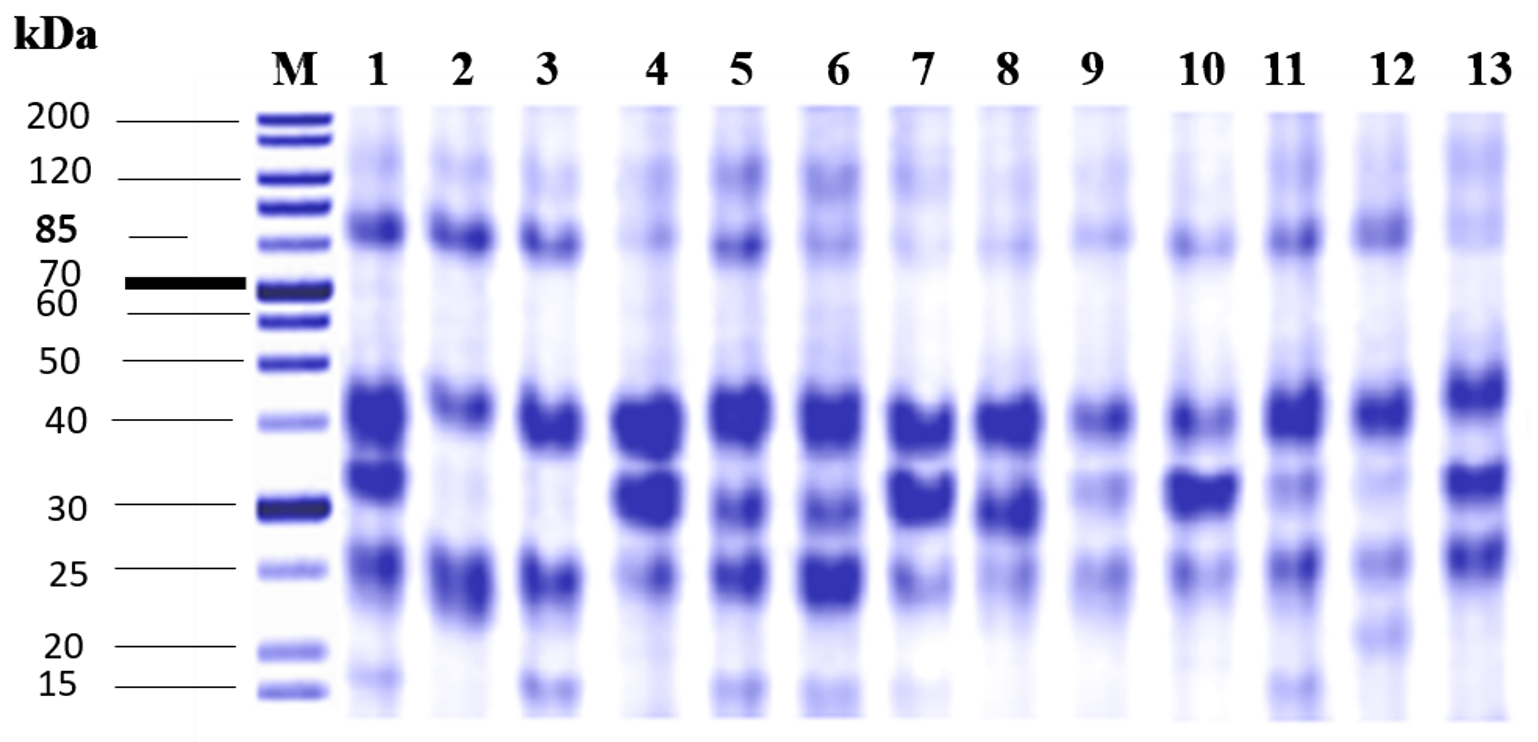

Supplement: Supplementary file 11 — Fig. S11. SDS‐PAGE (10%) for the recombinant choxAB expressed in E. coli BL21 (DE3) Rosetta. Each lane contained 50 µg total protein. M: protein ladder. Lanes 1–4: soluble fractions of cell lysate of recombinant cells induced at room temperature and 1 mM IPTG using M9, 2xTY, 5x LB, and LB growth media, respectively. Lanes 5–9: soluble fractions of cell lysate of recombinant cells induced at room temperature using 0.2, 0.4, 0.6, 0.8, and 1 mM IPTG, respectively. Lanes 10–12: soluble fractions of cell lysate of recombinant cells induced using 1mM IPTG at room temperature, 30 oC, and 37 oC, respectively. Lane 13: uninduced soluble fraction of cell lysate of recombinant cells. [file FEB4-11-2560-s007.tif]

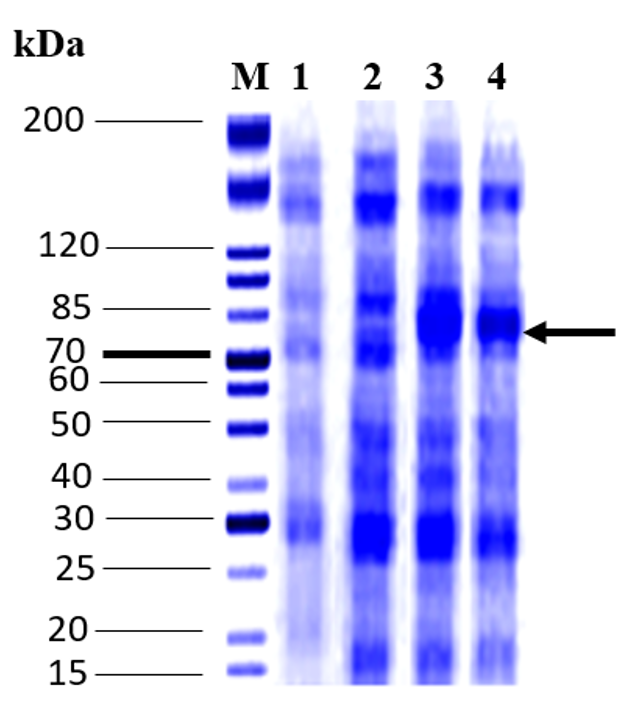

Supplement: Supplementary file 12 — Fig. S12. SDS‐PAGE (10%) for the recombinant choxAB expressed in E. coli BL21 (DE3) Rosetta. Each lane contained 50 µg total protein. M: protein ladder. Lanes (1–4): recombinant choxAB after solubilization with four solubilizing buffers namely C, F, E, and B, respectively. [file FEB4-11-2560-s001.tif]

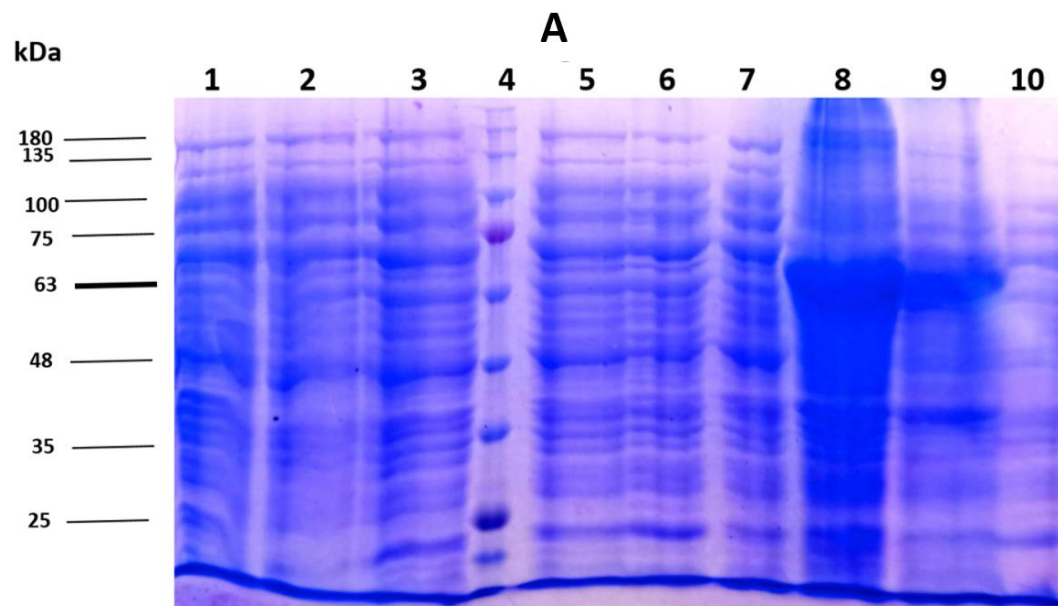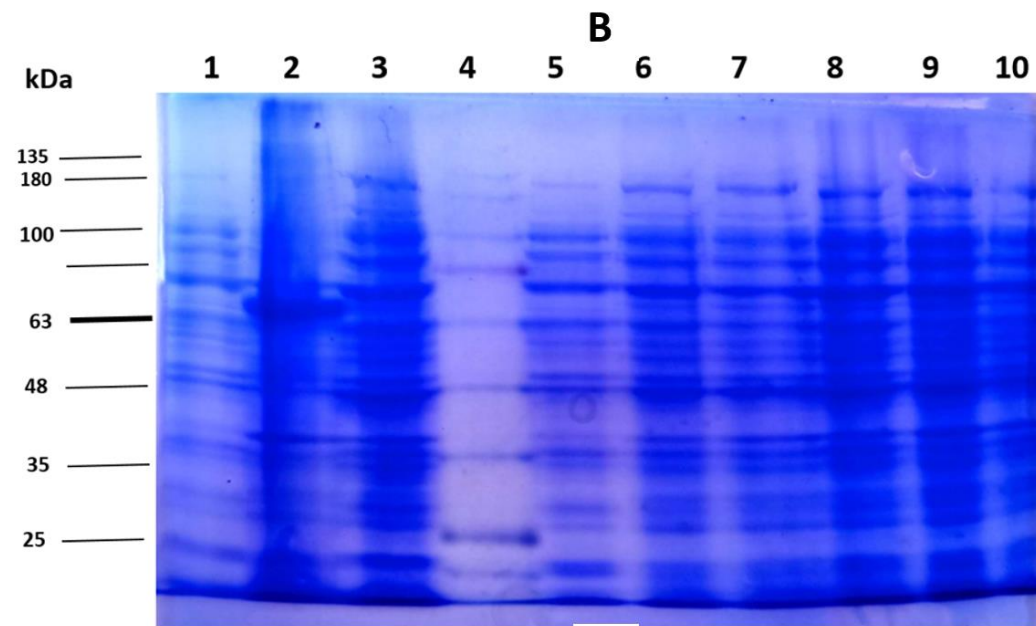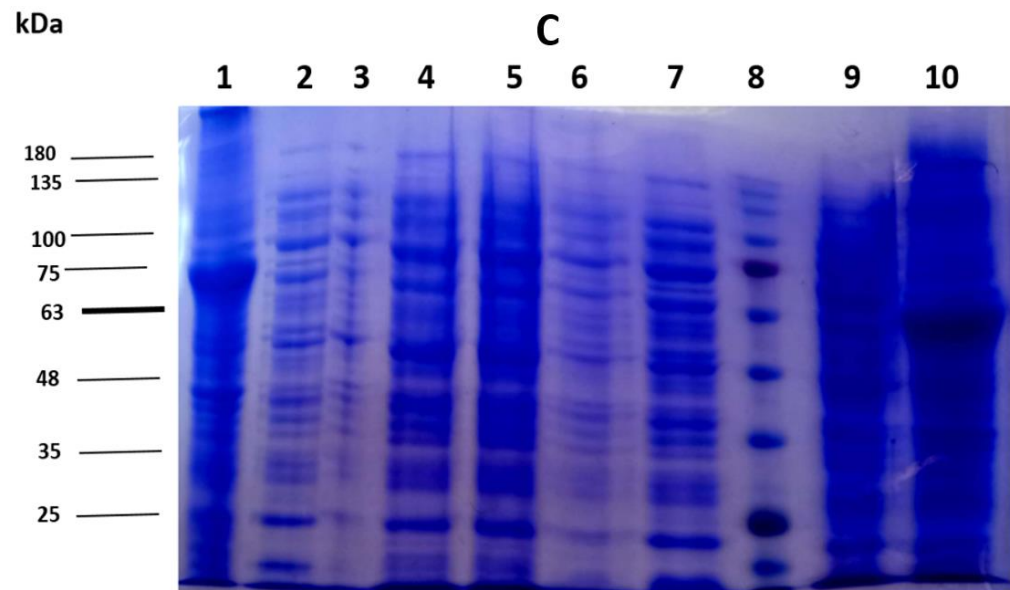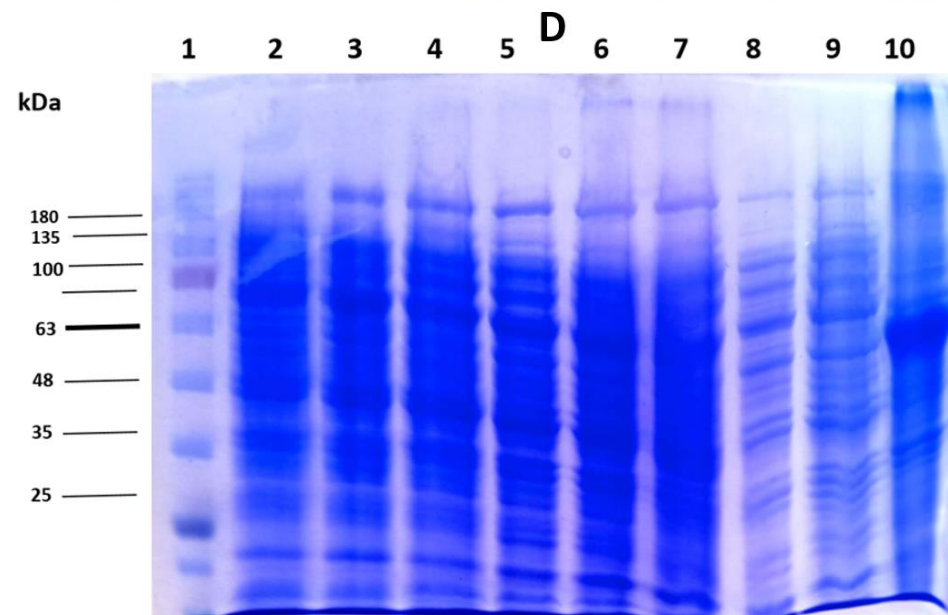

Supplement: Supplementary file 13 — Fig. S13. SDS‐PAGE (10%) for the recombinant choxAB expressed in E. coli BL21 (DE3) Rosetta where induction (with 1 mM IPTG) was performed in the presence of the following additives at the time of induction glycerol, ethanol, sorbitol, and glucose. Panel (A): lanes 1&2: soluble fractions of cell lysate of recombinant E.coli cells induced in the presence of 1% (v/v) ethanol and 3% (v/v) glycerol, respectively. Lane 3: soluble fraction of cell lysate of recombinant E.coli cells induced without any additives. Lane 4: Protein ladder. Lane 5 & 7: soluble fractions of cell lysate of recombinant E.coli cells induced in the presence of 0.2 M sorbitol and 15 mM glucose, respectively. Lanes 6 & 10: soluble fractions of cell lysate of uninduced recombinant E.coli cells. Lane 8 & 9: insoluble fractions of cell lysate of recombinant E.coli cells induced in the presence of 1% (v/v) ethanol and 0.2 M sorbitol, respectively. Panel (B): lanes 1 & 5 soluble fractions of cell lysate of uninduced recombinant E.coli cells, lane 2: insoluble fraction of cell lysate of recombinant E.coli cells induced in the presence of 0.3 M sorbitol, lane 3: soluble fraction of cell lysate of recombinant E.coli cells induced without any additives, lane 4: Protein ladder. Lanes 6–10: soluble fractions of cell lysate of recombinant E.coli cells induced in the presence of 5% (v/v) glycerol, 4% (v/v) glycerol, 3% (v/v) glycerol, 2% (v/v) glycerol, and 1% (v/v) glycerol, respectively. Panel (C): lanes 1 & 10: insoluble fractions of cell lysate of recombinant E.coli cells induced in the presence of 3% (v/v) glycerol and 4% (v/v) glycerol, respectively. Lanes 2 & 7: soluble fractions of cell lysate of uninduced recombinant E.coli cells, lanes 3–6: soluble fractions of cell lysate of recombinant E.coli cells induced in the presence of 0.4 M sorbitol, 0.3 M sorbitol, 0.2 M sorbitol, and 0.1 M sorbitol, respectively. Lane 8: Protein ladder. Lane 9: soluble fraction of cell lysate of recombinant E.coli cells induc [file FEB4-11-2560-s003.pdf]

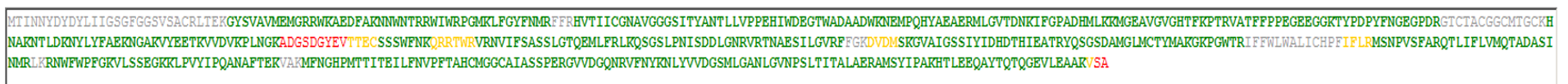

Supplement: Supplementary file 14 — Fig. S14. Concordance of choxAB amino acid sequence obtained via tryptic digestion and LC‐MS‐MS with that of Acinetobacter baumannii cholesterol oxidase (UniprotKB: A0A0E1FG24, 82.6% sequence coverage). Confidence of choxAB amino acids is indicated by color: green, good; yellow, mid; and red, low confidence. Gray letters represent amino acids not seen in a peptide of choxAB profiled by LC‐MS‐MS. [file FEB4-11-2560-s016.tif]
