# Supplementary material for: Cloning, expression, and in silico structural modeling of cholesterol oxidase of Acinetobacter sp. strain RAMD in E. coli
Source: FEBS Open Bio. 2021 Jul 31;11(9):2560–75. doi: 10.1002/2211-5463.13254 (PMC8409315; doi:10.1002/2211-5463.13254)
Supplement: Supplementary file 2 — Fig. S2. The primary amino acid sequence (556 amino acids) of the recombinant choxAB protein inferred from the translated open reading frame (1671 bp) of choxAB. Arrow indicates the start codon (M1); squares indicate the catalytic triad (E220, H380, and N514); star indicates the stop codon (TAA); rectangle indicates the conserved FAD‐binding sequence (GSGFGGSVSACRLTEKG). [file FEB4-11-2560-s010.pdf]

```
M T I N N Y D Y D Y L I I G S G F G G S V S A C R L T E K G Y S V A V M E M G R R W K A E D F A K
ATGACAATAAACAATTATGACTATGATTACCTGATTATTGGTTCAGGCTTTGGTGGTAGTGTTTCTGCTTGCCGTTTAACCGAAAAAGGCTACTCGGTTGCCGTAATGGAAATGGGGCGACGCTGGAAAGCTGAAGATTTTGCTAAA

N N W N T R R W I W R P G M K L F G Y F N M R F F R H V T I I C G N A V G G G S I T Y A N T L L V
AACAACTGGAATACTCGCCGTTGGATTTGGCGTCCGGGCATGAAACTTTTCGGTTATTTCAACATGCGTTTTTTTCGCCATGTCACCATTATTTGCGGTAATGCCGTAGGTGGCGGTTCAATTACCTATGCCAATACCCTACTGGTT

P P E H I W D E G T W A D A A D W K N E M P Q H Y A E A E R M L G V T D N K I F G P A D H M L K K
CCACCTGAACATATTTGGGATGAAGGCACGTGGGCAGATGCCGCGGACTGGAAAAATGAAATGCCTCAGCACTATGCTGAAGCAGAACGTATGCTTGGCGTGACTGATAATAAAATTTTCGGCCCTGCCGACCATATGCTAAAGAAA

M G E A V G V G H T F K P T R V A T F F P P E G E E G G K T Y P D P Y F N G E G P D R G T C T A C
ATGGGTGAAGCAGTCGGTGTTGGCCATACATTTAAACCTACTCGTGTTGCCACTTTCTTTCCACCGGAAGGTGAAGAAGGTGGGAAGACCTACCCTGACCCTTATTTTAACGGCGAAGGTCCAGACCGTGGCACTTGTACCGCATGT

G G C M T G C K H N A K N T L D K N Y L Y F A E K N G A K V Y E E T K V V D V K P L N G K A D G S
GGCGGTTGTATGACGGGTTGTAAACACAACGCAAAAAATACATTAGATAAAAACTATTTGTACTTTGCCGAAAAAAATGGCGCCAAGGTTTATGAAGAAACCAAAGTAGTTGATGTTAAACCACTCAATGGTAAAGCCGATGGCAGC

D G Y E V T T G C S S S W F N K Q R R T W R V R N V I F S A S S L G T Q E M L F R L K Q S G S L P
GACGGTTATGAAGTCACGACCGGATGTTCAAGCTCATGGTTTAACAAACAACGTCGTACTTGGCGAGTTCGTAATGTGATTTTCTCGGCATCTTCTTTAGGGACACAAGAGATGTTGTTCCGTTTAAAACAGTCTGGTTCTCTGCCA

N I S D D L G N R V R T N A E S I L G V R F F G K D V D M S K G V A I G S S I Y I D H D T H I E A
AATATTTCTGATGATTTGGGTAATCGGGTTCGTACCAATGCCGAGTCGATTTTAGGTGTTCGCTTTTTTGGTAAAGATGTCGACATGAGTAAAGGTGTTGCTATTGGTTCAAGTATTTACATTGATCATGACACTCACATTGAAGCG

T R Y Q S G S D A M G L M C T Y M A K G K P G W T R I F F W L W A L I C H P F I F L R M S N P V S
ACTCGCTACCAAAGTGGCTCAGATGCCATGGGCCTTATGTGTACTTACATGGCTAAAGGCAAACCGGGTTGGACGCGTATTTTCTTTTGGTTATGGGCACTCATTTGCCATCCATTTATTTTCCTTCGCATGAGTAATCCTGTCAGT

F A R Q T L I F L V M Q T A D A S I N M R L K R N W F W P F G K V L S S E G K K L P V Y I P Q A N
TTTGCACGTCAAACCTTAATTTTCCTTGTCATGCAAACTGCCGATGCCTCAATCAACATGCGCTTGAAACGTAACTGGTTCTGGCCTTTTGGTAAGGTGTTATCGAGCGAAGGTAAAAAGCTACCTGTTTATATTCCGCAGGCGAAT

A F T E K V A K M F N G H P M T T I T E I L F N V P F T A H C M G G C A I A S S P E R G V V D G Q
GCATTTACCGAAAAAGTTGCCAAAATGTTTAATGGCCACCCCATGACGACCATTACCGAAATTTTGTTTAACGTACCTTTTACAGCTCACTGTATGGGTGGTTGTGCAATTGCTTCAAGTCCAGAGCGAGGTGTGGTAGATGGACAA

N R V F N Y K N L Y V V D G S M L G A N L G V N P S L T I T A L A E R A M S Y I P A K H T L E E Q
AATCGGGTGTTTAACTATAAAAACTTATACGTTGTAGATGGTTCAATGTTAGGTGCAAATCTGGGGGTTAACCCAAGTCTCACCATTACAGCTCTAGCAGAACGTGCAATGTCTTATATTCCGGCTAAACATACTTTAGAAGAACAA

A Y T Q T H N E V L E A A K V S A
GCTTATACTCAAACTCATAATGAAGTTTTAGAAGCAGCAAAAGTATCTGCTTAA
```
