# Supplementary material for: Cloning, expression, and in silico structural modeling of cholesterol oxidase of Acinetobacter sp. strain RAMD in E. coli
Source: FEBS Open Bio. 2021 Jul 31;11(9):2560–75. doi: 10.1002/2211-5463.13254 (PMC8409315; doi:10.1002/2211-5463.13254)
Supplement: Supplementary file 15 — Table S1. Levels of cholesterol oxidase activity of choxAB from the cell lysate of induced recombinant E.coli cells under different cultural conditions. [file FEB4-11-2560-s012.docx]

Table S1 Levels of cholesterol oxidase activity of choxAB from the cell lysate of induced recombinant *E.coli* cells under different cultural conditions

| Culture condition | choxAB activity *(U/mL) |
| --- | --- |
| Uninduced recombinant *E.coli* cells | 0.00 |
| Induced recombinant *E.coli* cells under different induction temperatures | |
| 37 ^o^C | 0.000 |
| 30 ^o^C | 0.00004±0.00001 |
| RT | 0.008±0.0001 |
| Induced recombinant *E.coli* cells with different IPTG concentrations | |
| 0.2 mM | 0.00 |
| 0.4 mM | 0.00 |
| 0.6 mM | 0.0002±0.00001 |
| 0.8 mM | 0.00030±0.00002 |
| 1.0 mM | 0.008±0.0001 |
| Induced recombinant *E.coli* cells cultivated in different growth media | |
| 2xTY | 0.00002±0.000001 |
| LB | 0.008±0.0001 |
| M9 | 0.00 |
| 5xLB | 0.0004±0.000003 |

* cholesterol oxidase activity was determined in the soluble fraction of recombinant *E.coli* cells according to Richmond assay as mentioned in methods’section. All values were expressed as the average of three readings ± standard error.
