# Supplementary material for: Cloning, expression, and in silico structural modeling of cholesterol oxidase of Acinetobacter sp. strain RAMD in E. coli
Source: FEBS Open Bio. 2021 Jul 31;11(9):2560–75. doi: 10.1002/2211-5463.13254 (PMC8409315; doi:10.1002/2211-5463.13254)
Supplement: Supplementary file 16 — Table S2. Levels of solubilized cholesterol oxidase activity of choxAB in the insoluble fraction of the cell lysate of recombinant E.coli cells. [file FEB4-11-2560-s002.docx]

Table S2 Levels of solubilized cholesterol oxidase activity of choxAB in the insoluble fraction of the cell lysate of recombinant *E.coli* cells

| Solubilizing agent | choxAB activity *(U/mL) |
| --- | --- |
| Solubilization buffer B | 0.00 |
| Solubilization buffer C | 0.00 |
| Solubilization buffer E | 0.00 |
| Solubilization buffer F | 0.00 |
| SDS-KCl | 0.00 |

* cholesterol oxidase activity was determined in the insoluble fraction of recombinant *E.coli* cells after solubilization as mentioned in materials and methods
